# Human Milk Fortification and Necrotizing Enterocolitis in Very Low Birthweight Infants: State of Evidence and Systematic Review with Meta-Analysis

**DOI:** 10.3390/nu17213384

**Published:** 2025-10-28

**Authors:** Sarah M. Reyes, Tristen L. Paul, Jenelle Ferry

**Affiliations:** 1 Rev Bioscience, LLC, Boise, ID 83712, USA; 2 Pediatrix Medical Group Tampa Regional Practices, St. Joseph’s Women’s Hospital, Tampa, FL 33607, USA

**Keywords:** exclusive human milk diet, human milk fortifier, premature infant, very low birthweight, necrotizing enterocolitis

## Abstract

**Background:** Necrotizing enterocolitis (NEC) remains a leading cause of morbidity and mortality in very low birthweight (VLBW) infants. Human milk feeding and standardized feeding protocols are protective, but clinical practice varies, particularly in fortifier choice. Whether human milk-derived fortifiers reduce NEC risk compared with cow milk-derived fortifiers remains unclear. **Methods:** We conducted a systematic state-of-evidence review and meta-analysis, searching PubMed, Web of Science, and Scopus through July 2025. Eligible studies included RCTs and observational cohorts of VLBW infants comparing an exclusive human milk diet (EHMD) including human milk-derived fortifiers to cow milk-derived diets. Two reviewers independently screened and extracted data. Both RCTs and observational studies were included to evaluate consistency of effect estimates across designs and to account for heterogeneity in control group feeding practices. Pooled odds ratios (ORs) with 95% CIs were calculated using a Sidik–Jonkman random-effects model. Sensitivity analyses by study design and exclusion of infant formula from controls were performed. **Results:** Twenty studies (five RCTs, 15 observational; *n* = 6794 infants) met inclusion criteria, most enrolling infants born ≤1250 g. Compared with cow milk-containing diets, EHMD was associated with lower odds of Bell Stage ≥ 2 NEC (OR: 0.59; 95% CI: 0.42, 0.81; *p* < 0.001; *n* = 4625) and surgical NEC (OR: 0.43; 95% CI: 0.32, 0.58; *p* < 0.0001; *n* = 4754). In direct comparisons of fortifier type with a base diet of human milk, estimates suggested lower odds of Bell Stage ≥ 2 NEC by 35% (OR: 0.65; 95% CI: 0.44, 0.97; *p* = 0.03, *n* = 2102) and surgical NEC by 49% (OR: 0.51; 95% CI: 0.26, 0.98; *p* = 0.04; *n* = 1659) with human milk-derived fortifiers. Effect estimates were generally consistent across study designs, although precision and statistical significance varied. **Conclusions:** EHMD with human milk-derived fortifiers was associated with lower odds of medical and surgical NEC in VLBW infants, with most evidence from infants born ≤1250 g, reflecting current clinical use in the highest-risk population. Although the number and sample sizes of RCTs remain limited, the consistency of effect estimates across both RCTs and observational studies, together with significance of pooled analyses, strengthens confidence in these findings. Pragmatic and registry-based studies using standardized fortification protocols may provide the most efficient pathway to strengthen the evidence base.

## 1. Introduction

Necrotizing enterocolitis (NEC) is an inflammatory disease of the gastrointestinal tract, characterized by ischemia, enteric spillage, and/or necrosis of the intestine [1,2]. Primarily affecting premature infants, NEC remains a leading cause of morbidity and mortality in very low birthweight (VLBW) infants (<1500 g), with reported mortality rates of 20–30% and even higher rates among those requiring surgical intervention [1,3]. Lower birth weights and gestational ages are associated with a higher frequency and greater severity of disease [3,4,5]. The pathogenesis is not completely understood, but involves a complex interplay between intestinal immaturity, altered microbial colonization, and an exaggerated inflammatory response, often manifesting in the first few weeks or months of life [6]. Diagnosis is established using Bell’s staging criteria, which incorporate clinical, laboratory, and radiographic findings to classify disease severity from suspected (Stage 1) to advanced (Stage 3) cases with intestinal perforation [7,8].

Provision of human milk is associated with a reduced risk of NEC in VLBW infants [9]. It is currently thought that human milk protects against NEC through its bioactive components, which modulate inflammatory responses, support gastrointestinal maturation, and promote the development of a favorable microbiome [6,10,11]. However, VLBW infants have substantially higher nutritional requirements compared to term infants, particularly for protein, energy, fatty acids, minerals, and micronutrients [12]. While human milk provides optimal bioactive components, its native nutrient content cannot meet these elevated requirements within the limited feeding volumes that VLBW infants can safely tolerate [9]. Thus, the American Academy of Pediatrics (AAP) recommends fortification of human milk to support adequate growth in VLBW infants [13].

The demonstrated benefits of human milk for premature infant health have sparked increasing interest in human milk-derived fortifiers as an alternative to conventional cow milk-derived fortifiers. However, evidence regarding their comparative effectiveness in preventing NEC remains mixed. Several randomized controlled trials (RCTs) and observational studies have reported significant reductions in necrotizing enterocolitis (NEC) when VLBW infants receive an exclusive human milk diet (EHMD, human milk with human milk-based fortification) compared to diets containing cow milk-derived products [14,15,16,17]. Yet, two of the largest RCTs conducted to date found no differences in NEC among VLBW infants receiving human milk- versus cow milk-derived fortifiers as supplements to maternal or donor human milk [18,19].

This discordance between study results warrants careful examination. Existing RCTs face several methodological challenges, including insufficient statistical power and heterogeneity in fortification protocols, particularly regarding when fortification is initiated and how quickly it is advanced. Furthermore, substantial variation exists in control group feeding practices across studies. Some include infants receiving preterm formula, while others examine infants receiving exclusively human milk with cow milk-based fortification. Given the established dose–response relationship between human milk intake and improved clinical outcomes [20,21], this heterogeneity in baseline human milk exposure may significantly influence study findings.

Some previous systematic reviews, which have predominantly focused on RCT evidence, have concluded there is insufficient evidence for the effectiveness of an EHMD on NEC outcomes [22,23]. However, the systematic exclusion of observational evidence may overlook valuable real-world effectiveness data [17]. This perspective aligns with evolving approaches in pharmaceutical research, where real-world evidence increasingly complements RCT efficacy data to inform clinical decision-making and regulatory approvals [24].

Given limitations of previous systematic reviews, we sought to investigate the current state of evidence for the effect of human milk-derived fortifiers on NEC outcomes in premature or VLBW infants compared to cow milk-derived nutrition. We conducted a comprehensive systematic review and meta-analysis synthesizing both randomized and observational evidence comparing an EHMD with cow milk-derived nutrition, with and without infants receiving infant formula. This approach enabled examination of both efficacy and effectiveness while exploring potential sources of heterogeneity between study designs.

## 2. Methods

This systematic review and meta-analysis was conducted in accordance with the Preferred Reporting Items for Systematic reviews and Meta-Analyses (PRISMA) guidelines [25], and was registered on the OSF platform with registration ID *wcnfq*.

### 2.1. Search Strategy and Screening

PubMed (MEDLINE) was searched for relevant articles, with search queries including articles published in English through August 2024. We used a combination of controlled vocabulary and keywords to create search concepts for human milk and human milk fortifiers, premature and low birthweight infants, and NEC and feeding intolerance. The search was re-run in July 2025 alongside translated searches run in Web of Science and Scopus. The full search strategy is available in Table S1. Search results were consolidated, and duplicates were removed using Covidence (Veritas Health Innovation, Melbourne, Australia). Duplicates not detected by software were annotated and removed manually. All relevant articles were screened by two independent reviewers, blinded to each other’s decisions. Blinding was achieved through Covidence. Disagreements were resolved through consensus. A full list of studies excluded at the full text screening level is available in Table S2.

### 2.2. Study Selection Criteria

Any randomized controlled trial (RCT) or observational cohort study was eligible for inclusion if it investigated an EHMD vs. cow milk-based nutritional products and NEC in premature or low birthweight infants, defined as 37 weeks, 0 days of gestation or earlier and weighing ≤1500 g, respectively. An EHMD was defined as vat-pasteurized, human milk-derived human milk fortifiers added to a base diet of human milk, either mother’s own milk (MOM) and/or donor human milk (DHM). Infants in the control group must have received a diet containing cow milk-derived nutritional products, including a base diet of human milk (MOM and/or DHM) fortified with cow milk-derived human milk fortifiers and/or infant formula, as reported by study authors.

Exclusion criteria were the following: (1) studies of special populations, such as surgical infants; (2) studies without a comparator group receiving cow milk-derived nutritional products; (3) non-human studies (mechanistic or animal models); (4) case studies, (5) reviews, commentaries, letters to the editor, conference proceedings; (6) studies with no outcomes relevant to the review reported or available after contact (or attempted contact) with study authors. To avoid double-counting patients, we also excluded linked articles that would have resulted in duplicate entries per infant.

### 2.3. Data Extraction

Two reviewers independently collected data using a pre-set extraction form created for this review. Variables were collected at either the study level (e.g., article, subject and study design information) or group level (e.g., fortification- and outcome-related information). Outcome measures were captured as incidence and non-incidence, whereas all other variables were collected as reported by the article. Conflicts were resolved by discussion between reviewers or involvement of a third reviewer.

### 2.4. Outcome Measures and Data Harmonization

Our primary outcomes were medical NEC and surgical NEC (NEC requiring surgery), as defined by study authors. The definitions of medical NEC varied across studies, though most used the original or modified Bell Stage criteria [7,8] exclusively or in addition to other clinical symptoms to diagnose NEC. Some studies reported incidences of each Bell Stage of NEC or as collapsed variables. Herein, we report 2 collapsed variables: “any medical NEC”, representing the incidence of NEC regardless of Bell Stage, or “medical NEC (Bell Stage ≥ 2)”, representing the incidence of NEC Bell Stage 2 or higher. All RCTs reported Bell Stage ≥ 2, but only one reported NEC of all stages [18]. Therefore, for RCTs, we only report Bell Stage ≥ 2.

Outcome variables were re-coded, as needed, as indicator variables to harmonize results. In cases where outcome definitions varied, variables were re-coded, if possible, to allow for analyses. In cases where there were >1 intervention and/or control group, data were collapsed into a single intervention and control group. For continuous variables, mean and SD were extracted. In cases where median (IQR) was reported, the mean and SD were approximated using the following calculation based on Wan et al. [26]: mean equals the average of the two IQR values. The SD is the mean minus the lower value of the IQR times 1.33. The value of 1.33 is derived from the normal distribution curve. If the median was not close to the mean, the data were considered highly skewed, and the SD was considered unreliable. Therefore, the studies whose median was ≥10% lower than the mean were excluded from meta-analyses. Where data were missing or unavailable, we attempted to contact the corresponding author. Missing data were not imputed.

Exposure variables reflected enteral nutrition in the EHMD intervention and control diets (Table 1). The EHMD variable encompassed all exclusive human milk diet interventions utilizing human milk with human milk-derived fortifier, regardless of fortification protocols. Control diets contained cow milk-derived components and were stratified by presence or absence of commercial infant formula exposure. The CMD+F variable encompassed control groups in which at least some infants received formula. Studies with unclear formula feeding status were included only after contacting study authors for clarification. When authors could not be reached, the study was assumed to include formula feeding and was therefore excluded from head-to-head analyses. The CMD-F variable comprised infants exclusively fed human milk supplemented with cow milk-derived fortifier, enabling direct comparison between human and bovine fortification strategies in human milk-fed infants.

### 2.5. Risk of Bias Assessment

Risk of bias was independently assessed by two reviewers using separate tools based on the type and design of the study. Discrepancies between reviewers were resolved by discussion or consultation with a third reviewer.

Observational studies were assessed using the RoBNObs tool [27], developed and used in the 2020 Dietary Guidelines for Americans [28]. Risk was assessed according to the following domains: (1) confounding, (2) selection of participants, (3) classification of exposures, (4) departures from intended exposures, (5) missing data, (6) measurement of outcomes, and (7) selection of reported results. Each domain was assessed and scored using a rating of Low, Moderate, Serious, Critical, or No Information. Overall risk was determined by taking either the highest risk score across all domains or, for cases where there were multiple occurrences of a higher risk score across domains, downgraded based on evaluation and summation of individual domain risk scores. For example, where there were occurrences of ‘Moderate’ risk across four or more domains, the study was downgraded to receive an overall risk of ‘Serious’, and where there was an occurrence of ‘Serious” across three or more domains, the study was downgraded to receive an overall risk of ‘Critical’. A study was similarly downgraded if there were multiple occurrences of varying higher-risk assessments across domains. For example, two domains rated as ‘Serious’ and four domains rated as ‘Moderate’ would lead to a downgrade of ‘Critical’ risk.

Randomized trials were assessed using the Cochrane Risk of Bias (RoB2.0) tool [29]. Domains assessed included risk arising from (1) randomization, (2) deviation from intended intervention, (3) missing outcome data, (4) measurement of the outcome, and (5) selection of the reported result. Overall risk was rated as “low” where trials received a low-risk score across all five domains and as “some concerns” where at least one of five domains received a score of “some concerns”.

### 2.6. Statistical Analyses

We tested the associations between an EHMD and medical and surgical NEC compared to cow milk-containing diets, with and without infant formula (CMD+F and CMD-F, respectively). Pooled odds ratios (ORs) with 95% confidence intervals (CIs) were calculated in Stata 18 (StataCorp, LLC, College Station, TX, USA). Random-effects meta-analyses were performed using the Sidik–Jonkman estimator, followed by random-effects meta-regression to assess whether birthweight (BW) and gestational age (GA) moderated the effect sizes [30]. A random-effects approach was selected to account for between-study variability beyond that explained by BW and GA. Study heterogeneity was measured using the *I*^2^ statistic. Sensitivity analyses were performed separately for RCTs and observational cohort studies. Statistical significance was defined as 95% CIs not including the null and *p* < 0.05. Publication bias was assessed using funnel plots with the trim and fill method.

## 3. Results

### 3.1. Description of Included Studies

**Figure 1:**
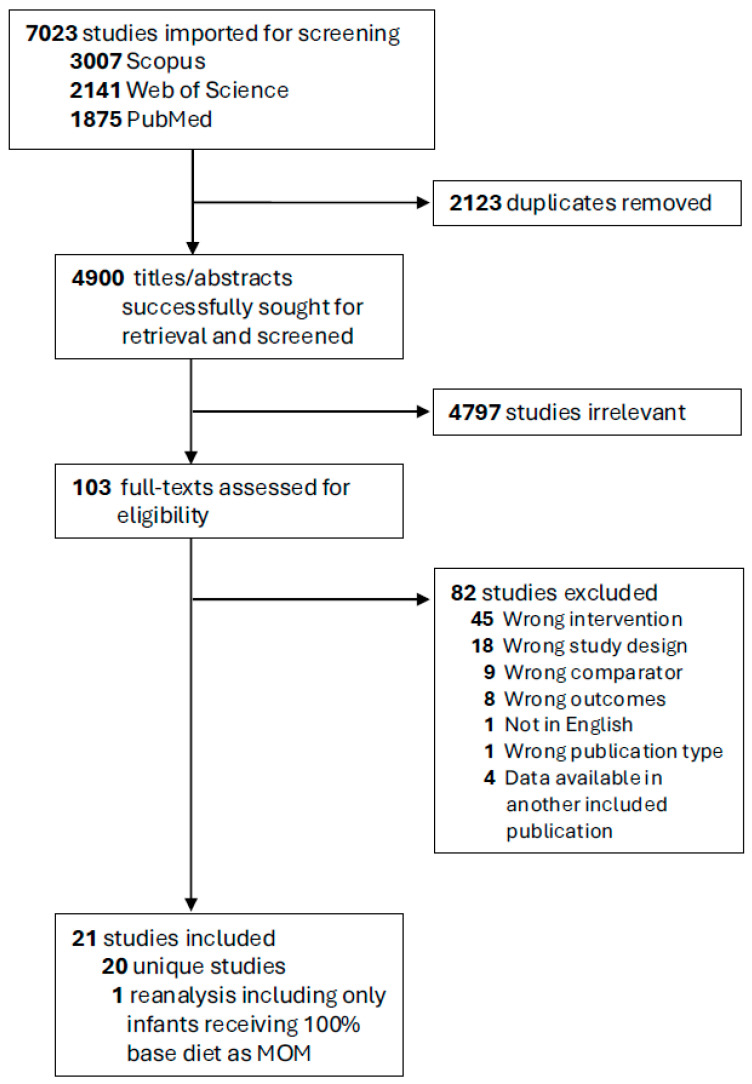
PRISMA flow diagram. —MOM, mother’s own milk.

In total, 7023 abstracts were identified and screened, 104 full texts were assessed for eligibility against selection criteria, and 20 were deemed appropriate for inclusion (Figure 1) [14,15,16,17,18,19,31,32,33,34,35,36,37,38,39,40,41,42,43,44]. The main reasons for study exclusion included wrong study design (e.g., systematic or narrative reviews, case reports); wrong intervention (e.g., intervention was not a vat-pasteurized human milk-derived fortifier); and wrong comparator (e.g., control diet did not consist of cow milk-based nutritional products).

Five randomized trials and 15 observational cohort studies were included in the final analyses (Table 2). Included studies were based exclusively in high-income countries in the Northern Hemisphere, with representative centers in the US, UK, Canada, Austria, and Sweden. Studies were a mix of single-center and multi-center designs (Table 2). The overall number of infants from the included studies totaled *n* = 6794. Of these, 47.7% (*n* = 3240) received an EHMD intervention consisting of a base diet of human milk with added vat-pasteurized human milk-derived fortifier; ~52.3% (*n* = 3554) received a diet containing at least some commercial cow milk-derived products. Reported birthweights ranged between 796 and 1361 g, and gestational age ranged from 25.5 to 29.8 weeks. The birth year of study infants ranged between 2004 and 2021.

Fortification initiation and advancement varied considerably within and across studies, as did the level of details reported (Table 2). Fortification for an EHMD began as early as 40 mL/kg/day and as late as 150 mL/kg/day. Older studies started EHMD fortification at 4 kcal/oz; more recent studies commonly initiated fortification at 6 kcal/oz.

Among the 20 included studies, 19 reported medical NEC, and 12 reported surgical NEC. Among studies that reported medical NEC, 14 defined it as Bell Stage ≥ 2 (Figure S1). Eleven studies had sufficient information to allow for head-to-head comparisons of human- vs. bovine-derived fortifiers (Figure S1) [16,17,18,19,31,35,40,42,43,44]. For two studies, Assad et al. and Sullivan et al., data for head-to-head comparisons were retrieved from study authors or companion publications [45]. Medical NEC was reported in all studies for which head-to-head comparisons were possible; six reported surgical NEC.

**Table 2:** Summary of Included Studies.

Publication and Location	Study Design	Participant Birth Year	Initial Eligibility Criteria	EHMD Fortification	Control Diet	Sample Size		
						EHMD	Control	Total
**Assad 2016 [16]** * US	Single center, retrospective cohort	2009–2014	GA ≤ 28 weeks BW ≤ 1500 g	Prolact+ H2MF^®^: 4 kcal/oz at 120–150 mL/kg/day *	MOM + cow milk-based fortifier @ 4 mL/kg/day at 120–150 mL/kg/day and/or preterm infant formula	87	206	293
**Bushati 2021 [31]** ** US	Single center, retrospective cohort	2018–2019	BW ≤ 1000 g	Prolact+ H2MF^®^ @ 6 kcal/oz at 60 mL/kg/day; 8 kcal/oz at 120 mL/kg/day; CR if weight gain <15 g/kg/day on Prolact+8 *	MOM + cow milk-based fortifier @ 2 kcal/oz at 80 mL/kg/day; 4 kcal/oz at 100 mL/kg/day (liquid protein fortifier, microlipids, and Neosure (formula) if inadequate growth)	15	49	64
**Carome 2021 [32]** US	Single center, retrospective cohort	2012–2017	ELBW infants born at or transferred to NICU within 24 h of birth	Prolact+ H2MF^®^ @ 4 kcal/oz at 80 mL/kg/day; then 6–8 kcal/oz if growth <15 g/kg/day	Preterm infant formula or MOM + cow milk-based fortifier @ 2 kcal/oz at 80 mL/kg/day; 4 kcal/oz 1 day after initiation; then 6 kcal/oz if growth <15 g/kg/day	127	179	306
**Colacci 2017 [33]** US	Single center, retrospective cohort	2011–2013	BW < 1000 g GA < 37 weeks	Not reported	MOM + cow milk-based fortifier and/or preterm formula	39	46	85
**Cristofalo 2013 [14]** US, Austria	Multicenter, RCT	2007–2008	BW 500–1250 g whose mothers did not intend to give milk	Prolact+ H2MF^®^ @ 40 mL/kg/day or 100 mL/kg/day	Preterm infant formula	29	24	53
**Eibensteiner 2019 [34]** Austria	Multicenter, retrospective cohort	2012–2018	BW < 1000 g and initiation of human milk fortification	Prolact+ H2MF^®^ @ 6 kcal/oz at 100 mL/kg/day or preterm infant formula	MOM + cow milk-based fortifier @ 4.4 kcal/oz at 100 mL/kg/day or preterm infant formula	96	96	192
**El-Fadeel 2022 [35]** US	Single center, retrospective cohort	2013–2018	BW < 1250 g who survived until discharge	Prolact+ H2MF^®^ @ 80–120 mL/kg/day	MOM + cow milk-based fortifier @ 80–100 mL/kg/day or preterm infant formula w/fortification at DOL 18 as needed	56	109	165
**Embleton 2023 [36]** UK	Multicenter, RCT	2017–2019	GA ≤ 28 6/7 weeks and <72 h age	Prolact+ H2MF^®^ @ 6 kcal at 150 mL/kg/day	MOM + preterm formula @ 150 mL/kg/day	63	63	126
**Hair 2016 [46]** US	Multicenter, retrospective cohort	2006–2014	BW < 1250 g	Prolact+ H2MF^®^ @ 60 mL/kg/day or 100–120 mL/kg/day	MOM+ cow milk-based fortifier and/or preterm infant formula	819	768	1587
**Hanford 2021 [38]** US	Single center, retrospective cohort	2016–2018	BW < 1100 g GA < 30 weeks	Prolact+ H2MF^®^ @ 4–8 kcal/oz at 80 mL/kg/day depending on growth	MOM + cow milk-based fortifier @ 80 mL/kg/day or preterm infant formula depending on growth	53	36	89
**Harris 2024 [39]** US	Single center, retrospective cohort	Not reported	BW < 1250 g GA < 32 weeks	4 kcal/oz @ 40 mL/kg/day	Not reported	105	96	201
**Herrmann 2014 [40]** US	Single center, retrospective cohort	2004–2012	GA < 33 weeks	Prolact+ H2MF^®^ start/advancement not reported	MOM + cow milk-based fortifier start/advancement not reported	199	443	642
**Huston 2018 [41]** US	Single center, retrospective cohort	2007–2015	BW 500–1250 g	Prolact+ H2MF^®^ @ 4 kcal/oz at 40–50 mL/kg/day or 80–100 mL/kg/day; 6 kcal/oz at variable volumes or 130 mL/kg/day, then 8 kcal/oz if fluid restricted to <145 mL/kg/day of enteral feedings or poor growth	MOM+ cow milk-based fortifier @ 4 kcal/oz at 80–100 mL/kg/day; 6 kcal/oz at variable volumes (or 130 mL/kg/day), then 7 kcal/oz Enfacare (formula) if fluid restricted to <145 mL/kg/day of enteral feedings or poor growth	127	252	379
**Jensen 2024 [19]** Sweden	Multicenter, RCT	2019–2021	GA 22 + 0–27 + 6 weeks and ability to maintain intervention until PMA 34 weeks	Targeted fortification w/Prolacta Humavant+6 before 100 mL/kg/day (protein goal 4.0–4.5 g/kg/day, then gradually decreased as infant approaches term equivalent)	Targeted fortification w/cow milk-based fortifier before <100 mL/kg/day (protein goal 4.0–4.5 g/kg/day, then gradually decreased as infant approaches term equivalent)	115	113	228
**O’Connor 2018 [18]** Canada	Multicenter, RCT	2014–2016	BW < 1250 g and parent consent to donor human milk; enteral feeding started within 14 d of birth	Prolact+ H2MF^®^ 4 kcal/oz at 100 mL/kg/day; 6 kcal at 140 mL/kg/day, then 8 kcal if weight gain <15 g/kg/day after achieving full feeds (160 mL/kg/day)	MOM + cow milk-based fortifier @ 2 kcal/oz at 100 mL/kg/day; 4 kcal/oz at 140 mL/kg/day, then 6 kcal/oz if weight gain <15 g/kg/day after achieving full feeds	64	63	127
**Sato 2020 [42]** ** US	Single center, retrospective cohort	2012–2018	BW 1000–1499 g	Prolact+ H2MF^®^ @ 4 kcal/oz at 90 mL/kg/day	MOM+ cow milk-based fortifier @ 4 kcal/oz at 90 mL/kg/day	265	134	399
**Sullivan 2010 [15]** *** US, Austria	Multicenter, RCT	2007–2008	BW 500–1250 g	Prolact+ H2MF^®^ @ 40 mL/kg/day or 100 mL/kg/day *	MOM + cow milk-based fortifier @ 100 mL/kg/day or preterm infant formula	138	69	207
**Swanson 2023 [17]** US	Multicenter, retrospective cohort	Not reported	BW < 1000–≤ 1500 g GA < 28–< 32 weeks	Prolact+ H2MF^®^ @ 60 mL/kg/day or 80 mL/kg/day with fortification goal of 26–28 kcal/oz or 26–32 kcal/oz	Cow milk-based fortifier @ 60 mL/kg/day or 80 mL/kg/day with fortification goal of 26–28 kcal/oz or 26–32 kcal/oz	511	526	1037
**Tetarbe 2024 [43]** US	Single center, retrospective cohort	2015–2016, 2020–2021	VLBW infants fed MOM or DHM fortified with CMDF or HMDF and ability to maintain intervention until PMA 34 weeks	Prolact+ H2MF^®^ @ 80 mL/kg/day and human milk caloric fortifier (2 kcal/oz) when infants tolerated 120 mL/kg/day of enteral feeds and were off PN	MOM/DHM + cow milk-based fortifier @ 80 mL/kg/day	57	64	121
**Wickland 2022 [44]** US	Single center, retrospective cohort	2013–2016	BW ≤ 1250 g GA ≤ 33 weeks	Prolact+ H2MF^®^ @ 4 kcal/oz @ 80 mL/kg/day or Prolact+ H2MF^®^ @ 6 kcal/oz at 80 mL/kg/day fortified to 24 kcal/oz	MOM/DHM + cow milk-based fortifier @ 80 mL/kg/day fortified to 24 kcal/oz	275	218	493

* Values presented here may differ slightly from those originally reported, reflecting clarifications obtained directly from the study authors. ** All ELBW infants also received Ultimate Flora Baby Probiotics (RenewLife, Palm Harbor, FL). *** For head-to-head fortifier comparisons, data were extracted from Lucas 2020 [45].

### 3.2. Associations of an EHMD with Infant Medical NEC

#### 3.2.1. Associations of an EHMD vs. Any Cow Milk-Containing Diet (CMD+F) and Medical NEC

Any medical NEC was reported in 19 studies [14,15,16,17,18,19,31,32,33,34,35,38,39,40,41,42,43,44,46]. Overall, compared to a diet containing cow milk products (CMD+F), an EHMD was associated with a 42% reduction in any medical NEC (OR: 0.58; 95% CI, 0.43, 0.79; *p* < 0.001; *I*^2^ 39.4%; 6708 participants) (Figure S2).

**Figure 2:**
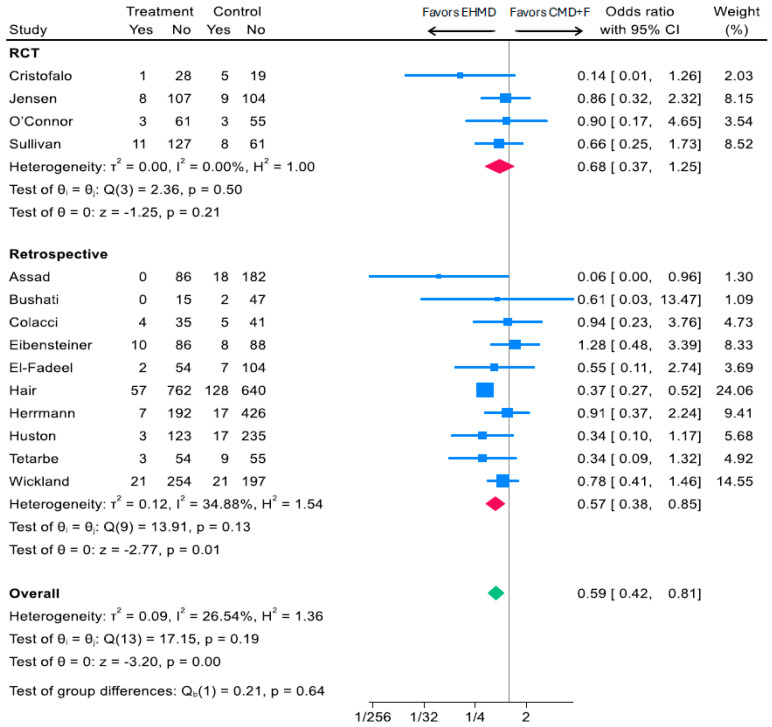
Associations of an EHMD vs. cow milk-containing diet (CMD+F) with medical NEC (Bell Stage ≥ 2) among low birthweight infants (*n* = 4625). Forest plots were generated based on random-effects meta-analysis using the Sidik–Jonkman method for between-study variance estimation. Blue squares represent study-specific odds ratios, with the size proportional to the study weight. Horizontal lines indicate 95% CI. Red diamonds indicate pooled estimates for RCTs and retrospective studies, and the green diamond indicates the overall pooled estimate—CI, confidence interval; CMD+F, cow milk-containing diet that may include infant formula; EHMD, exclusive human milk diet; NEC, necrotizing enterocolitis.

Fourteen studies including four RCTs and 10 observational cohorts reported medical NEC as Bell Stage ≥ 2 [14,15,16,18,19,31,33,34,35,40,41,43,44,46]. Overall, compared to a diet containing cow milk products (CMD+F), an EHMD was associated with a 41% reduction in medical NEC (Bell Stage ≥ 2) (OR: 0.59; 95% CI, 0.42, 0.81; *p* < 0.001; *I*^2^ 26.54%; 4625 participants) (Figure 2). Among RCTs, an EHMD was associated with a 32% reduction in Bell Stage ≥ 2 medical NEC (OR: 0.68; 95% CI, 0.37, 1.25; *p* = 0.21; *I*^2^ 0%; 610 participants) (Figure 2). Among observational cohorts, an EHMD was associated with a 43% reduction in Bell Stage ≥ 2 medical NEC (OR: 0.57; 95% CI, 0.38, 0.85; *p* = 0.001; *I*^2^ 34.88%; 4015 participants) (Figure 2).

#### 3.2.2. Head-to-Head Comparison of Human vs. Cow Milk-Based Fortifiers and Medical NEC

Any medical NEC was reported in 11 studies [15,16,17,18,19,31,35,40,42,43,44]. Overall, in a head-to-head comparison of fortifiers, an EHMD was associated with a 33% reduction in any medical NEC compared to CMD-F (OR: 0.67; 95% CI, 0.44, 1.03; *p* = 0.07; *I*^2^ 19.94%; 2805 participants) (Figure S3).

**Figure 3:**
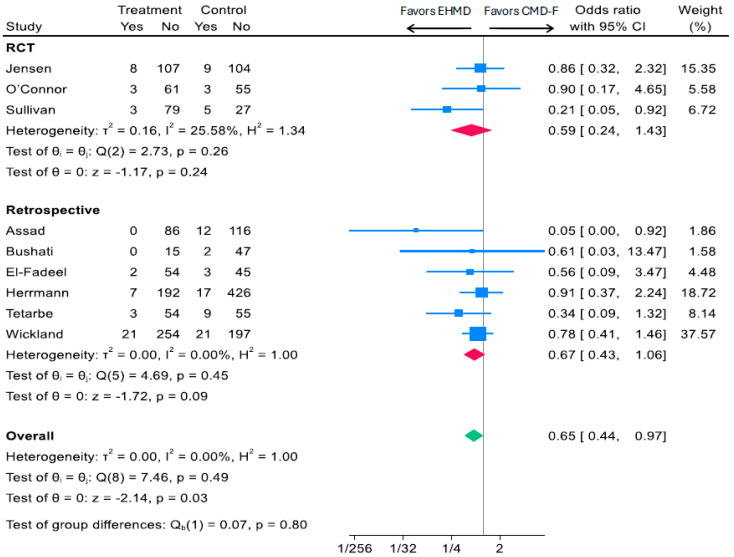
Associations of EHMD vs. cow milk-derived fortifiers added to a base diet of human milk (CMD-F) with medical NEC (Bell Stage ≥ 2) among low birthweight infants (*n* = 2102). Forest plots were generated based on random-effects meta-analysis using the Sidik–Jonkman method for between-study variance estimation. Blue squares represent study-specific odds ratios, with the size proportional to the study weight. Horizontal lines indicate 95% CI. Red diamonds indicate pooled estimates for RCTs and retrospective studies, and the green diamond indicates the overall pooled estimate—CI, confidence interval; CMD-F, cow milk fortifiers added to human milk; EHMD, exclusive human milk diet; NEC, necrotizing enterocolitis.

For medical NEC (Bell Stage ≥ 2), nine studies including three RCTs and seven observational cohorts reported a head-to-head comparison of human milk-derived vs. cow milk-based fortifiers added to a base diet of human milk [15,16,18,19,31,35,40,43,44]. Overall, compared to CMD-F, an EHMD was associated with a 35% reduction in medical NEC (Bell Stage ≥ 2) (OR: 0.65, 95% CI, 0.44, 0.97; *p* = 0.03; *I*^2^ 0%; 2102 participants) (Figure 3). A similar effect size was seen for RCTs (OR: 0.59, 95% CI, 0.24, 1.43; *p* = 0.24; *I*^2^ 25.58%; 464 participants) and observational cohorts (OR: 0.67, 95% CI, 0.43, 1.06; *p* = 0.09; *I*^2^ 0%; 1638 participants) (Figure 3).

### 3.3. Associations of an EHMD with Infant Surgical NEC

#### 3.3.1. EHMD vs. Any Cow Milk Diet (CMD+F) and Surgical NEC

**Figure 4:**
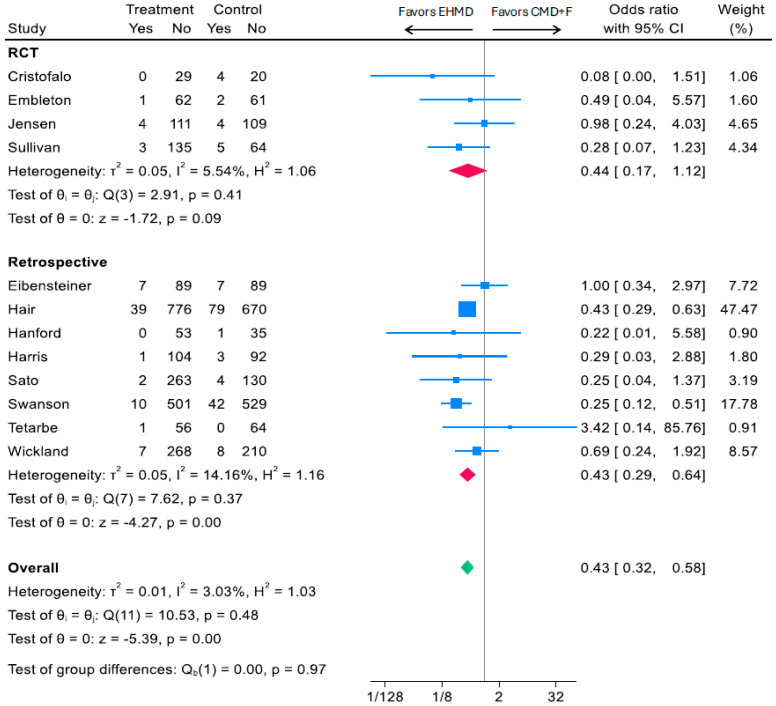
Associations of an EHMD vs. cow milk-containing diet (CMD+F) with surgical NEC among low birthweight infants (*n* = 4754). Forest plots were generated based on random-effects meta-analysis using the Sidik–Jonkman method for between-study variance estimation. Blue squares represent study-specific odds ratios, with the size proportional to the study weight. Horizontal lines indicate 95% CI. Red diamonds indicate pooled estimates for RCTs and retrospective studies, and the green diamond indicates the overall pooled estimate—CI, confidence interval; CMD+F, cow milk-containing diet that may include infant formula; EHMD, exclusive human milk diet; NEC, necrotizing enterocolitis.

Surgical NEC was reported in 12 studies; four studies were RCTs and eight were observational cohorts [14,15,17,19,34,36,38,39,42,43,44,46]. In pooled analyses, we found that an EHMD was associated with a 57% reduction in surgical NEC compared to a CMD+F (OR: 0.43; 95% CI, 0.32, 0.58; *I*^2^ 3.03%; *p* < 0.0001; 4754 participants) (Figure 4). Effect sizes were similar across study type, with a 56% reduction among RCTs (OR: 0.44; 95% CI, 0.17, 1.12; *I*^2^ 5.54%; *p* = 0.09; 614 participants) and 57% reduction among observational cohorts (OR: 0.44; 95% CI, 0.29, 0.64; *I*^2^ 14.16%; *p* = 0.0001; 4140 participants) (Figure 4).

#### 3.3.2. Head-to-Head Comparison of Human vs. Cow Milk-Based Fortifiers and Surgical NEC

**Figure 5:**
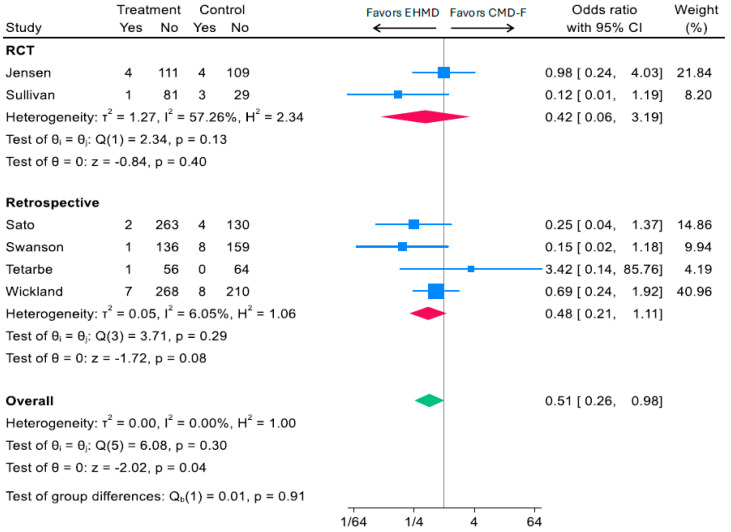
Associations of EHMD vs. cow milk-derived fortifiers added to a base diet of human milk (CMD-F) with surgical NEC among low birthweight infants (*n* = 1659). Forest plots were generated based on random-effects meta-analysis using the Sidik–Jonkman method for between-study variance estimation. Blue squares represent study-specific odds ratios, with the size proportional to the study weight. Horizontal lines indicate 95% CI. Red diamonds indicate pooled estimates for RCTs and retrospective studies, and the green diamond indicates the overall pooled estimate—CI, confidence interval; CMD-F, cow milk fortifiers added to human milk; EHMD, exclusive human milk diet; NEC, necrotizing enterocolitis.

Six studies including two RCTs and four observational cohorts investigated a head-to-head comparison of human milk-derived vs. cow milk-derived fortifiers [15,17,19,42,43,44]. In pooled analyses, an EHMD was associated with a 49% reduction in odds of developing surgical NEC compared to a CMD-F (OR: 0.51; 95% CI, 0.26, 0.98; *I*^2^ 0.00%%; *p* = 0.04; 1659 participants) (Figure 5). Effect sizes were similar across study type, with a 52% reduction among RCTs (OR: 0.42; 95% CI, 0.06, 3.19; *I*^2^ 4657.26%; *p* = 0.40; 342 participants) and 52% reduction among observational cohorts (OR: 0.48; 95% CI, 0.21, 1.11; *I*^2^ 6.05%; *p* = 0.08; 1317 participants) (Figure 5).

### 3.4. Quality Assessment and Publication Bias

Visual inspection of funnel plots indicated no signs of publication bias (Figure S4). For quality assessment, four of five RCTs were rated as “low risk” of bias [14,15,18,19], and one was rated as “some concerns” [36] (Table S3). Of the observational studies, five studies were rated as “critical risk” [17,33,40,41,42,47], seven as “serious risk” [31,32,35,43,46], and another four studies as “moderate risk” [16,34,38,44] (Table S4).

Overall, RCTs received low risk scores across all domains, owing to the sufficient reporting of methods, statistical analyses plans, and participant- and outcome-level data and the relative completeness of such data (Table S3). An elevated risk of bias was found for only domain 1 (risk arising from randomization) and was caused primarily by a failure to report participant allocation concealment [36]. It is worth noting, however, that risk of bias assessment tools for randomized studies are limited because they exclude thorough exposure assessment—a potential confounding factor. For example, in the trial by Jensen et al. [19], fortification was initiated in the intervention group at a significantly lower volume than in the control group. This plausibly may lead to differences in NEC risk between groups, even when human milk is the base diet for both groups. However, the RoB2 tool (nor any other tool to assess RCT quality) does not capture this level of detail to the exposure nutritional intervention.

The representation of overall higher risk ratings across observational studies was due to most studies receiving elevated risk ratings across individual domains (Table S4). For example, some studies had biased participant selection procedures due to practicalities within the clinical practice [41,47]. Others failed to adjust for differences in baseline or confounding factors, including differences in the nutrition intervention received by study infants [42]. The sum of these domains (e.g., four-plus “moderate risk” domains with at least one “serious risk” domain) resulted in no observational studies receiving an overall “low risk” assessment. All observational studies in this review were rated as being at moderate risk or higher for domain 1 (bias due to confounding) due to the inherent confounding present in studies with this design, combined with the manner in which the RoBNObs risk assessment tool has been constructed to assess this domain. Though comprehensive, the tool makes it difficult for observational studies to obtain low risk ratings, as the expectation is comparable to that for a well-designed RCT. For example, the publication by Swanson et al. [17] received an overall critical risk rating due to confounding inherently not being controllable, and this is acknowledged by the authors. Because this study was a review of multiple studies by a panel of experts, certain domains (4, 5, 6) were rated as having “No information”, increasing the overall risk score. Similarly to domain 1, domain 7 (bias in selection of reported results) largely comprised studies obtaining a moderate risk rating. This is primarily due to lack of pre-registration of study protocols. Only two of the 16 studies provided proof of pre-registration [34,40]. Domain 5 (missing data) generally received lower risk ratings across studies when compared to other domains, owing to the relative completeness of data or the adjustment for missing data, where applicable.

## 4. Discussion

### 4.1. Key Findings

This systematic review with meta-analysis investigated the state of evidence on human milk-derived fortifiers and NEC outcomes among VLBW infants. Compared with diets containing any cow milk-derived fortifiers or formula, an exclusive human milk diet (EHMD) including human milk-derived fortifiers was associated with ~30–40% lower odds of Bell Stage ≥ 2 medical NEC and ~50% lower odds of in surgical NEC. In direct comparisons of human milk-derived vs. cow milk-derived fortifiers with a base diet of human milk (i.e., no infant formula in controls), an EHMD was associated with a 33% lower odds of Bell Stage ≥ 2 medical NEC and 50% lower odds of surgical NEC. In total, 2422 infants were included in medical NEC analyses and 1715 infants in surgical NEC analyses. Effect estimates were similar across study types, and differences in statistical significance between RCTs and observational studies likely reflect sample size and power limitations rather than conflicting results, a phenomenon well recognized in rare disease research where even small outcome differences can shift statistical significance [48]. These findings highlight the potential clinical relevance of human milk-derived fortifiers for NEC outcomes and underscore the importance of interpreting results in terms of effect estimates and precision, not only *p*-values [49].

### 4.2. RCTs, Real-World Data, and Variability in Clinical Practice

NEC incidence in VLBW infants has declined over the past two decades. Real-world evidence from the Vermont Oxford Network (VON), a large multicenter clinical registry, showed a decline in medical NEC from 5.3% to 3.0% between 2006 and 2017 (*p* < 0.0001) [3]. This decline coincided with increased adoption of human milk feeding and standardized feeding protocols in NICUs [50] and is further supported by evidence that human milk feeding is associated with lower NEC risk than formula feeding [51,52].

Despite these improvements, fortification practices continue to vary considerably across NICUs. This may reflect more than scientific uncertainty: VLBW infants are both the population at greatest risk for NEC and the group for whom the AAP most strongly recommends fortification to meet elevated nutrient needs [13]. These dual pressures, supporting growth and minimizing NEC risk, help explain persistent variability in practice and underscore the importance of clarifying the evidence base.

Existing RCTs of human milk-derived fortifiers have produced mixed results, but these trials enrolled small numbers of infants (53–228 total) at a time when NEC incidence was already declining. As such, they were underpowered to detect clinically meaningful effects as statistically significant. Importantly, point estimates from RCTs remain directionally consistent with those from larger observational studies [17,37], suggesting that null results reflect inadequate power rather than absence of benefit.

Large-scale observational cohorts therefore provide essential real-world evidence, capturing outcomes across diverse NICU settings with sample sizes that would be cost-prohibitive in traditional RCTs. While such studies are inherently more vulnerable to residual confounding, the magnitude and consistency of effect estimates across multiple cohorts strengthen confidence that the protective association observed in our analyses is real. Taken together, our pooled analyses suggest that the apparent discrepancies between RCTs and observational data reflect methodological limitations rather than a true difference in the clinical effect.

These findings also help contextualize prior systematic reviews, which have reported mixed conclusions, reflecting varying inclusion criteria. A Cochrane review by Premkumar et al. (2019), including only a single RCT, concluded there was insufficient evidence for EHMD effectiveness [23]. By contrast, broader reviews that included formula-exposed control groups suggested protective associations of magnitude similar to ours: Anthan et al. (2020) found a 62% reduction in NEC (RR 0.28, 95% CI 0.15–0.95) and an 87% reduction in surgical NEC (RR 0.13, 95% CI 0.02–0.67), while Grace et al. (2021) reported a 53% reduction in NEC (RR 0.47, 95% CI 0.22–0.98) [53,54]. More recently, Galis et al. (2024) restricted inclusion to trials directly comparing fortifier type (human vs. cow milk-derived) with a base diet of human milk and found no statistically significant difference in NEC [22]. However, this analysis was limited to 681 infants in total and included RCTs that were underpowered to detect NEC. While prior reviews illustrate that the current evidence base is constrained by small RCTs and heterogeneous designs, our synthesis of the state of evidence in its entirety highlights a directionally consistent signal.

Only two randomized controlled trials have directly compared human milk- vs. cow milk-derived fortifiers in infants receiving a base diet of human milk [15,19]. Although the number of available studies is limited, the estimated effect sizes for surgical NEC were similar in magnitude and direction to those observed in overall pooled analyses. This consistency indicates that the apparent protective association of an EHMD is not solely attributable to comparisons against formula exposure but extends to fortifier type. Nevertheless, additional studies with larger sample sizes are warranted to strengthen the evidence base and confirm these findings.

### 4.3. Cost-Effectiveness Considerations

Cost is frequently cited as a barrier to adopting human milk-derived fortifiers, given higher acquisition costs compared with cow milk-derived fortifiers. Multiple cost–utility analyses conducted in the U.S. and internationally suggest that human milk-derived fortifiers are economically favorable considering reductions in NEC, surgical interventions, prolonged NICU stays, and long-term sequelae [55,56,57,58]. Under these assumptions, higher upfront product costs may be offset by downstream savings from avoided morbidity and disability. While results are context-dependent, integrating economic evidence with clinical outcomes provides a more comprehensive assessment of the potential value of fortification strategies.

Although NEC is now relatively rare within individual NICUs, at a population level, it remains a significant driver of mortality, morbidity, and cost, with U.S. incidence rates exceeding those reported in many other developed countries [3,59]. This discrepancy helps explain why some centers may perceive NEC as a limited problem, while at a national and international level, it continues to represent both important health and economic burdens. This contrast between local rarity and national burden highlights the importance of considering both institutional and population-level perspectives when evaluating strategies to prevent NEC.

### 4.4. Ethical Challenges and Evolving Clinical Practice

Nutrition trials in VLBW infants face unique ethical and logistical challenges. Randomization is the gold standard for establishing causality, but our review highlights substantial observational evidence of reduced NEC risk when VLBW infants are fed an EHMD. This raises important questions about equipoise, including clinical equipoise, which reflects genuine uncertainty within the expert medical community about whether one intervention is superior; and individual equipoise, which reflects the uncertainty, or lack thereof, of the treating clinician [60].

At the level of clinical equipoise, uncertainty plausibly persists because existing RCTs are few, underpowered, and in some cases non-significant. By contrast, individual equipoise maybe be fragile: many clinicians perceive human milk-derived fortifiers as safer and hesitate to introduce cow milk-based fortifiers [61,62,63,64]. This was evident in the Jensen trial, where fortification was initiated on average at ~10 mL/kg/day later in infants randomized to cow milk-derived fortifiers than in those receiving human milk-derived fortifiers (*p* = 0.01) [19]. Such deviations illustrate how clinical judgment and perceived safety could undermine protocol adherence, a challenge noted more broadly in trial methodology where bias and operational pressures may compromise study integrity [60].

Illustrative power calculations suggest that, assuming a baseline NEC incidence of 3.0%, detecting a 30–40% reduction in NEC with 80% statistical power would require enrollment of roughly 5000–9000 infants, numbers that render traditional randomized trials impractical. These constraints, compounded by heterogeneity in fortification timing, advancement protocols, and baseline human milk exposure, highlight both the limitations of existing trials and the challenges of conducting new ones [64,65,66]. Pragmatic and registry-based randomized designs embedded within established neonatal networks may therefore offer the most viable pathway to generate robust evidence while maintaining individualized care [67,68,69]

### 4.5. Future Directions

Several priorities emerge from this synthesis. First, fortification protocols, including timing, advancement, and concentration, must be standardized and consistently reported to better enable cross-study comparisons. Current heterogeneity complicates interpretation and may obscure true effects. Second, NEC risk varies markedly by birthweight: 12% among infants born at 501–750 g; 9.2% risk for those born at 751–1000 g; 5.7% risk for those born at 1001–1250 g; and 3.3% risk for infants born at 1251–1500 g [4]. Aggregating across all VLBW infant categories may obscure meaningful differences.

Third, network meta-analysis may eventually clarify the comparative effectiveness of different strategies, but current limitations in study quality and sample size caution against premature conclusions using this type of analysis [70]. Emerging interventions such as human milk cream supplementation show early promise and should be studied within standardized feeding protocols [71,72]. Finally, feeding intolerance is a potential early marker of NEC, yet remains inconsistently defined and reported [73]. A standardized definition (e.g., ≥24 h interruption as clinically meaningful) would improve comparability and support development of preventive strategies and may be a more practical intermediate outcome to measure in smaller trials.

### 4.6. Strengths and Limitations

The primary strength of this meta-analysis is the comprehensive inclusion of both RCTs and observational studies, providing the most complete state-of-evidence evaluation of fortification strategies for VLBW infants to date. Nonetheless, methodological constraints inherent to nutritional meta-analyses limit the certainty of conclusions. Standard risk of bias assessments for RCTs do not capture clinically relevant differences in fortification timing, advancement, or concentration, which may influence outcomes but remain unaccounted for in bias scoring. Similarly, residual confounding cannot be excluded, as few studies adjust for established factors independently associated with risk of or protection from NEC, such as race or early postnatal steroid use. Interpretation is further complicated by the fact that NEC incidence has declined in recent years, a trend attributed to greater availability of donor human milk, adoption of standardized feeding protocols, transition from powdered to liquid fortifiers, and adoption of probiotics along with improved antibiotic stewardship. Another limitation is that most studies enrolled infants ≤1250 g, but few reported outcomes stratified by birthweight, making it difficult to determine how well infants born <750 g were represented. Some pooled analyses included a small number of studies or infants, which limits the accuracy and precision of effect estimates, particularly for rare outcomes such as surgical NEC. Even so, we previously reported an individual participant data meta-analysis in this subgroup that demonstrated significant reductions in NEC with EHMD including human milk-derived fortifiers [74]. Taken together, these findings suggest that the protective association of EHMD is robust in infants ≤1250 g and extends to those <750 g, although more harmonized reporting, fortification practices, robust adjustment for confounders, and adequately powered trials capable of detecting differences in rare outcomes such as NEC would further strengthen confidence in the evidence base.

## 5. Conclusions

In this state-of-evidence and systematic review with meta-analysis, an EHMD was associated with significantly lower odds of both medical and surgical NEC among VLBW infants weighing ≤1250 g. Compared to diets containing cow milk-based nutrition, an EHMD was associated with ~30–40% reduction in medical NEC (Bell Stage ≥ 2) and ~50% reduction in surgical NEC. The consistency of effect sizes across study designs, despite variations in statistical significance, suggests that the benefits of human milk-derived fortifiers are clinically meaningful. While methodological challenges and ethical considerations may limit future randomized trials, the collective evidence supports the protective role of an EHMD compared to cow milk-based nutrition. Moving forward, standardization of fortification protocols, feeding advancement strategies, and reporting metrics will be crucial for strengthening the evidence base. As NICUs continue to evolve their feeding practices, careful documentation of both protocols and outcomes will help clarify the optimal approach to nutrition for this vulnerable population. These findings have important implications for clinical practice and underscore the need for continued research to refine our understanding of fortification strategies that maximize benefits while minimizing risks in VLBW infant care.

## Figures and Tables

**Table 1:** Exposure variables reflecting enteral nutrition in intervention and control diets.

Variable	Definition	Base Diet	Fortifier
EHMD	Exclusive human milk diet	Human milk	Vat-pasteurized human milk-derived fortifier
CMD+F	Cow milk-containing diet that may include formula	Varied by study, including formula or human milk as base diet	Cow milk-derived fortifier or infant formula
CMD-F	Cow milk-containing diet excluding formula	Human milk	Cow milk-derived fortifier

## Data Availability

No new data were created or analyzed in this study.

## Supplementary Materials

The following supporting information can be downloaded at: https://www.mdpi.com/article/10.3390/nu17213384/s1, Table S1. Search strategy for PubMed, Table S2. Excluded studies (*n* = 82), Table S3. Risk of bias assessment for RCTs, Table S4. Risk of bias assessment for observational studies, Figure S1. Summary of included studies, feeding group comparisons, and NEC outcomes, Figure S2. Associations of an EHMD vs. cow milk-containing diet with medical NEC among low birthweight infants (*n* = 6708), Figure S3. Associations of EHMD vs. cow milk-derived fortifiers added to a base diet of human milk with medical NEC among low birthweight infants (*n* = 2805), Figure S4. Funnel plots by study design and NEC outcome.

## References

[1] Neu J., Walker W.A. (2011). Necrotizing enterocolitis. N. Engl. J. Med..

[2] Robinson J.R., Rellinger E.J., Hatch L.D., Weitkamp J.H., Speck K.E., Danko M., Blakely M.L. (2017). Surgical necrotizing enterocolitis. Semin. Perinatol..

[3] Han S.M., Hong C.R., Knell J., Edwards E.M., Morrow K.A., Soll R.F., Modi B.P., Horbar J.D., Jaksic T. (2020). Trends in incidence and outcomes of necrotizing enterocolitis over the last 12 years: A multicenter cohort analysis. J. Pediatr. Surg..

[4] Fitzgibbons S.C., Ching Y., Yu D., Carpenter J., Kenny M., Weldon C., Lillehei C., Valim C., Horbar J.D., Jaksic T. (2009). Mortality of necrotizing enterocolitis expressed by birth weight categories. J. Pediatr. Surg..

[5] Hull M.A., Fisher J.G., Gutierrez I.M., Jones B.A., Kang K.H., Kenny M., Zurakowski D., Modi B.P., Horbar J.D., Jaksic T. (2014). Mortality and management of surgical necrotizing enterocolitis in very low birth weight neonates: A prospective cohort study. J. Am. Coll. Surg..

[6] Singh D.K., Miller C.M., Orgel K.A., Dave M., Mackay S., Good M. (2022). Necrotizing enterocolitis: Bench to bedside approaches and advancing our understanding of disease pathogenesis. Front. Pediatr..

[7] Bell M.J., Ternberg J.L., Feigin R.D., Keating J.P., Marshall R., Barton L., Brotherton T. (1978). Neonatal necrotizing enterocolitis. Therapeutic decisions based upon clinical staging. Ann. Surg..

[8] Walsh M.C., Kliegman R.M. (1986). Necrotizing enterocolitis: Treatment based on staging criteria. Pediatr. Clin. N. Am..

[9] Parker M.G., Stellwagen L.M., Noble L., Kim J.H., Poindexter B.B., Puopolo K.M., Section on Breastfeeding, Committee on Nutrition, Committee on Fetus and Newborn (2021). Promoting Human Milk and Breastfeeding for the Very Low Birth Weight Infant. Pediatrics.

[10] Autran C.A., Kellman B.P., Kim J.H., Asztalos E., Blood A.B., Spence E.C.H., Patel A.L., Hou J., Lewis N.E., Bode L. (2018). Human milk oligosaccharide composition predicts risk of necrotising enterocolitis in preterm infants. Gut.

[11] Bode L. (2018). Human Milk Oligosaccharides in the Prevention of Necrotizing Enterocolitis: A Journey From in vitro and in vivo Models to Mother-Infant Cohort Studies. Front. Pediatr..

[12] Kleinman R.E., Greer F.R. (2019). Pediatric Nutrition.

[13] Meek J.Y., Noble L., Section on Breastfeeding (2022). Policy Statement: Breastfeeding and the Use of Human Milk. Pediatrics.

[14] Cristofalo E.A., Schanler R.J., Blanco C.L., Sullivan S., Trawoeger R., Kiechl-Kohlendorfer U., Dudell G., Rechtman D.J., Lee M.L., Lucas A. (2013). Randomized trial of exclusive human milk versus preterm formula diets in extremely premature infants. J. Pediatr..

[15] Sullivan S., Schanler R.J., Kim J.H., Patel A.L., Trawöger R., Kiechl-Kohlendorfer U., Chan G.M., Blanco C.L., Abrams S., Cotten C.M. (2010). An exclusively human milk-based diet is associated with a lower rate of necrotizing enterocolitis than a diet of human milk and bovine milk-based products. J. Pediatr..

[16] Assad M., Elliott M.J., Abraham J.H. (2016). Decreased cost and improved feeding tolerance in VLBW infants fed an exclusive human milk diet. J. Perinatol..

[17] Swanson J.R., Becker A., Fox J., Horgan M., Moores R., Pardalos J., Pinheiro J., Stewart D., Robinson T. (2023). Implementing an exclusive human milk diet for preterm infants: Real-world experience in diverse NICUs. BMC Pediatr..

[18] O’Connor D.L., Kiss A., Tomlinson C., Bando N., Bayliss A., Campbell D.M., Daneman A., Francis J., Kotsopoulos K., Shah P.S. (2018). Nutrient enrichment of human milk with human and bovine milk-based fortifiers for infants born weighing <1250 g: A randomized clinical trial. Am. J. Clin. Nutr..

[19] Jensen G.B., Domellof M., Ahlsson F., Elfvin A., Naver L., Abrahamsson T. (2024). Effect of human milk-based fortification in extremely preterm infants fed exclusively with breast milk: A randomised controlled trial. EClinicalMedicine.

[20] Brownell E.A., Matson A.P., Smith K.C., Moore J.E., Esposito P.A., Lussier M.M., Lerer T.J., Hagadorn J.I. (2018). Dose-response Relationship Between Donor Human Milk, Mother’s Own Milk, Preterm Formula, and Neonatal Growth Outcomes. J. Pediatr. Gastroenterol. Nutr..

[21] Zhang B., Xiu W., Dai Y., Yang C. (2020). Protective effects of different doses of human milk on neonatal necrotizing enterocolitis. Medicine.

[22] Galis R., Trif P., Mudura D., Mazela J., Daly M.C., Kramer B.W., Diggikar S. (2024). Association of Fortification with Human Milk versus Bovine Milk-Based Fortifiers on Short-Term Outcomes in Preterm Infants-A Meta-Analysis. Nutrients.

[23] Premkumar M.H., Pammi M., Suresh G. (2019). Human milk-derived fortifier versus bovine milk-derived fortifier for prevention of mortality and morbidity in preterm neonates. Cochrane Database Syst. Rev..

[24] Thokagevistk K., Coppo C., Rey L., Carelli A., Diez V., Vaselenak S., Oliveira L., Patel A., Sicari E., Ramos T. (2024). Real-World Evidence to Reinforce Clinical Trial Evidence in Health Technology Assessment: A Critical Review of Real-World Evidence Requirements from Seven Countries and Recommendations to Improve Acceptance. J. Mark. Access Health Policy.

[25] Page M.J., McKenzie J.E., Bossuyt P.M., Boutron I., Hoffmann T.C., Mulrow C.D., Shamseer L., Tetzlaff J.M., Akl E.A., Brennan S.E. (2021). The PRISMA 2020 statement: An updated guideline for reporting systematic reviews. BMJ.

[26] Wan X., Wang W., Liu J., Tong T. (2014). Estimating the sample mean and standard deviation from the sample size, median, range and/or interquartile range. BMC Med. Res. Methodol..

[27] Nutrition Evidence Systematic Review Team (U.S. Department of Agriculture) (2019). Risk of Bias for Nutrition Observational Studies (RoB-NObs) Tool.

[28] 2025 Dietary Guidelines Advisory Committee (U.S. Department of Health and Human Services) (2024). Scientific Report of the 2025 Dietary Guidelines Advisory Committee: Advisory Report to the Secretary of Health and Human Services and Secretary of Agriculture.

[29] Sterne J.A.C., Savović J., Page M.J., Elbers R.G., Blencowe N.S., Boutron I., Cates C.J., Cheng H.-Y., Corbett M.S., Eldridge S.M. (2019). RoB 2: A revised tool for assessing risk of bias in randomised trials. BMJ.

[30] Sidik K., Jonkman J.N. (2005). Simple Heterogeneity Variance Estimation for Meta-Analysis. J. R. Stat. Soc. Ser. C Appl. Stat..

[31] Bushati C., Chan B., Harmeson Owen A., Woodbury A., Yang M., Fung C., Lechtenberg E., Rigby M., Baserga M. (2021). Challenges in Implementing Exclusive Human Milk Diet to Extremely Low-Birth-Weight Infants in a Level III Neonatal Intensive Care Unit. Nutr. Clin. Pract..

[32] Carome K., Rahman A., Parvez B. (2021). Exclusive human milk diet reduces incidence of severe intraventricular hemorrhage in extremely low birth weight infants. J. Perinatol..

[33] Colacci M., Murthy K., DeRegnier R.O., Khan J.Y., Robinson D.T. (2017). Growth and Development in Extremely Low Birth Weight Infants After the Introduction of Exclusive Human Milk Feedings. Am. J. Perinatol..

[34] Eibensteiner F., Auer-Hackenberg L., Jilma B., Thanhaeuser M., Wald M., Haiden N. (2019). Growth, Feeding Tolerance and Metabolism in Extreme Preterm Infants under an Exclusive Human Milk Diet. Nutrients.

[35] El-Fadeel H., Velumula P., Lulic-Botica M., Natarajan G., Thomas R., Botica G., Bajaj M. (2022). Effect of an exclusive human milk diet on feeding tolerance in preterm infants. J. Perinatol..

[36] Embleton N.D., Sproat T., Uthaya S., Young G.R., Garg S., Vasu V., Masi A.C., Beck L., Modi N., Stewart C.J. (2023). Effect of an Exclusive Human Milk Diet on the Gut Microbiome in Preterm Infants: A Randomized Clinical Trial. JAMA Netw. Open.

[37] Hair A.B., Peluso A.M., Hawthorne K.M., Perez J., Smith D.P., Khan J.Y., O’Donnell A., Powers R.J., Lee M.L., Abrams S.A. (2016). Beyond Necrotizing Enterocolitis Prevention: Improving Outcomes with an Exclusive Human Milk-Based Diet. Breastfeed. Med..

[38] Hanford J., Mannebach K., Ohler A., Patten M., Pardalos J. (2021). Rates of Comorbidities in Very Low Birth Weight Infants Fed an Exclusive Human Milk Diet Versus a Bovine Supplemented Diet. Breastfeed. Med..

[39] Harris L., Lewis S., Vardaman S. (2024). Exclusive Human Milk Diets and the Reduction of Necrotizing Enterocolitis. Adv. Neonatal Care.

[40] Herrmann K., Carroll K. (2014). An exclusively human milk diet reduces necrotizing enterocolitis. Breastfeed. Med..

[41] Huston R.K., Markell A.M., McCulley E.A., Gardiner S.K., Sweeney S.L. (2018). Improving Growth for Infants </=1250 Grams Receiving an Exclusive Human Milk Diet. Nutr. Clin. Pract..

[42] Sato R., Malai S., Razmjouy B. (2020). Necrotizing Enterocolitis Reduction Using an Exclusive Human-Milk Diet and Probiotic Supplementation in Infants With 1000-1499 Gram Birth Weight. Nutr Clin Pract.

[43] Tetarbe M., Chang M.R., Barton L., Cayabyab R., Ramanathan R. (2024). Economic and Clinical Impact of Using Human Milk-Derived Fortifier in Very Low Birth Weight Infants. Breastfeed. Med..

[44] Wickland J., Wade C., Micetic B., Meredith K., Martin G. (2022). A Retrospective Analysis of the Effects of an Exclusively Human Milk Protein Diet on Neonatal Feeding Tolerance. Am. J. Perinatol..

[45] Lucas A., Boscardin J., Abrams S.A. (2020). Preterm Infants Fed Cow’s Milk-Derived Fortifier Had Adverse Outcomes Despite a Base Diet of Only Mother’s Own Milk. Breastfeed. Med..

[46] Hair A.B., Bergner E.M., Lee M.L., Moreira A.G., Hawthorne K.M., Rechtman D.J., Abrams S.A., Blanco C.L. (2016). Premature Infants 750-1,250 g Birth Weight Supplemented with a Novel Human Milk-Derived Cream Are Discharged Sooner. Breastfeed. Med..

[47] Huston R.K., Markell A.M., McCulley E.A., Pathak M., Rogers S.P., Sweeney S.L., Dolphin N.G., Gardiner S.K. (2014). Decreasing Necrotizing Enterocolitis and Gastrointestinal Bleeding in the Neonatal Intensive Care Unit. ICAN Infant Child Adolesc. Nutr..

[48] Mitani A.A., Haneuse S. (2020). Small Data Challenges of Studying Rare Diseases. JAMA Netw. Open.

[49] Baker M. (2016). Statisticians issue warning over misuse of P values. Nature.

[50] Parker M.G., Greenberg L.T., Edwards E.M., Ehret D., Belfort M.B., Horbar J.D. (2019). National Trends in the Provision of Human Milk at Hospital Discharge Among Very Low-Birth-Weight Infants. JAMA Pediatr..

[51] Altobelli E., Angeletti P.M., Verrotti A., Petrocelli R. (2020). The Impact of Human Milk on Necrotizing Enterocolitis: A Systematic Review and Meta-Analysis. Nutrients.

[52] Schanler R.J., Shulman R.J., Lau C. (1999). Feeding strategies for premature infants: Beneficial outcomes of feeding fortified human milk versus preterm formula. Pediatrics.

[53] Ananthan A., Balasubramanian H., Rao S., Patole S. (2020). Human Milk-Derived Fortifiers Compared with Bovine Milk-Derived Fortifiers in Preterm Infants: A Systematic Review and Meta-Analysis. Adv. Nutr..

[54] Grace E., Hilditch C., Gomersall J., Collins C.T., Rumbold A., Keir A.K. (2021). Safety and efficacy of human milk-based fortifier in enterally fed preterm and/or low birthweight infants: A systematic review and meta-analysis. Arch. Dis. Child. Fetal Neonatal Ed..

[55] Ganapathy V., Hay J.W., Kim J.H. (2012). Costs of necrotizing enterocolitis and cost-effectiveness of exclusively human milk-based products in feeding extremely premature infants. Breastfeed. Med..

[56] Hampson G., Roberts S.L.E., Lucas A., Parkin D. (2019). An economic analysis of human milk supplementation for very low birth weight babies in the USA. BMC Pediatr..

[57] Scholz S.M., Greiner W. (2019). An exclusive human milk diet for very low birth weight newborns-A cost-effectiveness and EVPI study for Germany. PLoS ONE.

[58] Igarashi A., Reyes S.M., Mizuno K. (2025). Cost-utility analysis of an exclusive human milk diet for very low birthweight infants in Japan. J. Med. Econ..

[59] Battersby C., Santhalingam T., Costeloe K., Modi N. (2018). Incidence of neonatal necrotising enterocolitis in high-income countries: A systematic review. Arch. Dis. Child. Fetal Neonatal Ed..

[60] Alexander J.H. (2022). Equipoise in Clinical Trials: Enough Uncertainty in Whose Opinion?. Circulation.

[61] Henderson G., Craig S., Brocklehurst P., McGuire W. (2009). Enteral feeding regimens and necrotising enterocolitis in preterm infants: A multicentre case-control study. Arch. Dis. Child. Fetal Neonatal Ed..

[62] McKeown R.E., Marsh T.D., Amarnath U., Garrison C.Z., Addy C.L., Thompson S.J., Austin J.L. (1992). Role of delayed feeding and of feeding increments in necrotizing enterocolitis. J. Pediatr..

[63] Berseth C.L., Bisquera J.A., Paje V.U. (2003). Prolonging small feeding volumes early in life decreases the incidence of necrotizing enterocolitis in very low birth weight infants. Pediatrics.

[64] Fenin A., Newman J.C., Taylor S.N. (2020). Very low birth weight infants receive full enteral nutrition within 2 postnatal weeks. J. Perinatol..

[65] Salas A.A., Gunawan E., Nguyen K., Reeves A., Argent V., Finck A., Carlo W.A. (2023). Early Human Milk Fortification in Infants Born Extremely Preterm: A Randomized Trial. Pediatrics.

[66] Huston R., Lee M., Rider E., Stawarz M., Hedstrom D., Pence M., Chan V., Chambers J., Rogers S., Sager N. (2020). Early fortification of enteral feedings for infants <1250 grams birth weight receiving a human milk diet including human milk based fortifier. J. Neonatal Perinatal Med..

[67] Mathes T., Buehn S., Prengel P., Pieper D. (2018). Registry-based randomized controlled trials merged the strength of randomized controlled trails and observational studies and give rise to more pragmatic trials. J. Clin. Epidemiol..

[68] Zuidgeest M.G.P., Goetz I., Groenwold R.H.H., Irving E., van Thiel G.J.M.W., Grobbee D.E., GetReal Work Package 3 (2017). Series: Pragmatic trials and real world evidence: Paper 1. Introduction. J. Clin. Epidemiol..

[69] Clancy C.M., Margolis P.A., Miller M. (2013). Collaborative networks for both improvement and research. Pediatrics.

[70] Rouse B., Chaimani A., Li T. (2017). Network meta-analysis: An introduction for clinicians. Intern. Emerg. Med..

[71] Hair A.B., Blanco C.L., Moreira A.G., Hawthorne K.M., Lee M.L., Rechtman D.J., Abrams S.A. (2014). Randomized trial of human milk cream as a supplement to standard fortification of an exclusive human milk-based diet in infants 750-1250 g birth weight. J. Pediatr..

[72] Knake L.A., King B.C., Gollins L.A., Hurst N.M., Hagan J., Ford S.L., Hair A.B. (2020). Optimizing the Use of Human Milk Cream Supplement in Very Preterm Infants: Growth and Cost Outcomes. Nutr. Clin. Pract..

[73] Fanaro S. (2013). Feeding intolerance in the preterm infant. Early Hum. Dev..

[74] Reyes S.M., Moore J.B., Lee M.L., Ferry J., Elliott M.J. (2023). Associations of an Exclusive Human Milk Diet with Morbidity and Mortality in ELBW Infants Born Weighing <750 Grams: An Individual Participant Data Meta-analysis. Neonatol. Today.

