# Supplementary material for: Human Milk Fortification and Necrotizing Enterocolitis in Very Low Birthweight Infants: State of Evidence and Systematic Review with Meta-Analysis
Source: Nutrients. 2025 Oct 28;17(21):3384. doi: 10.3390/nu17213384 (PMC12609769; doi:10.3390/nu17213384)
Supplement: Supplementary file 1 [file nutrients-17-03384-s001.zip › nutrients-3950813-supplementary/Figure S1 ListofStudies.pdf]

Study	Design	Control Diet		Outcome			Notes
		CMBD+F	CMBD-F	All NEC	2+ NEC	Surgical NEC	
Assad 2015[16]*	Cohort	☑	☑		■		Authors confirmed data for head-to-head comparisons
Bushati 2021[31]	Cohort	☐	☑	■	■		
Carome 2020[32]	Cohort	☑	☐	■			
Colacci 2017[33]	Cohort	☑	☐		■		
Cristofalo 2013[14]	RCT	☑	☐		■	■	
Eibensteiner 2019[34]	Cohort	☑	☐		■	■	
El-Fadeel 2022[35]	Cohort	☑	☑		■		Two control groups: exclusive formula and MOM+bovine fortifier. These groups were collapsed in primary analyses. Only the MOM+bovine fortifier group was included in the head-to-head comparisons
Embleton 2023[36]	RCT	☑	☐			■	
Hair 2016[46]	Cohort	☑	☐		■	■	
Hanford 2021[38]	Cohort	☑	☐	■		■	
Harris 2024[39]	Cohort	☑	☐	■		■	
Herrmann 2014[40]	Cohort	☐	☑		■		
Huston 2018[41]	Cohort	☑	☐	■	■		Ultimately, removed Huston 2014 due to substantial overlap with Huston 2018
Jensen 2023[19]	RCT	☐	☑		■	■	
O'Connor 2018[18]	RCT	☐	☑	■	■		
Sato 2020[42]	Cohort	☐	☑	■		■	
Sullivan 2010[15]**	RCT	☑	☑		■	■	Head-to-head from Lucas 2020.[45] Note this excluded donor human milk and only included infants who received 100% MOM fortified with human- vs. cow milk-derived fortifier
Swanson 2023[17]	Cohort	☑	☑	■		■	Author confirmed that UVA was the only institution that didn't have formula in the control group, thus data for that institution was used in head-to-head comparisons
Tetarbe 2024[43]	Cohort	☐	☑		■	■	
Wickland 2022[44]	Cohort	☐	☑		■	■	

Figure S1. Summary of included studies, feeding group comparisons, and NEC outcomes. This table lists all studies included in the meta-analyses by design (RCT or observational cohort), comparator type, and reported NEC outcomes (any medical NEC, Bell Stage ≥2, and surgical NEC). Shaded cells indicate inclusion of the outcome in analyses. Notes summarize study-specific considerations such as overlapping datasets, collapsed control groups, or author clarifications for head-to-head comparisons.
