# Supplementary material for: Human Milk Fortification and Necrotizing Enterocolitis in Very Low Birthweight Infants: State of Evidence and Systematic Review with Meta-Analysis
Source: Nutrients. 2025 Oct 28;17(21):3384. doi: 10.3390/nu17213384 (PMC12609769; doi:10.3390/nu17213384)
Supplement: Supplementary file 1 [file nutrients-17-03384-s001.zip › nutrients-3950813-supplementary/Figure S2.pdf]

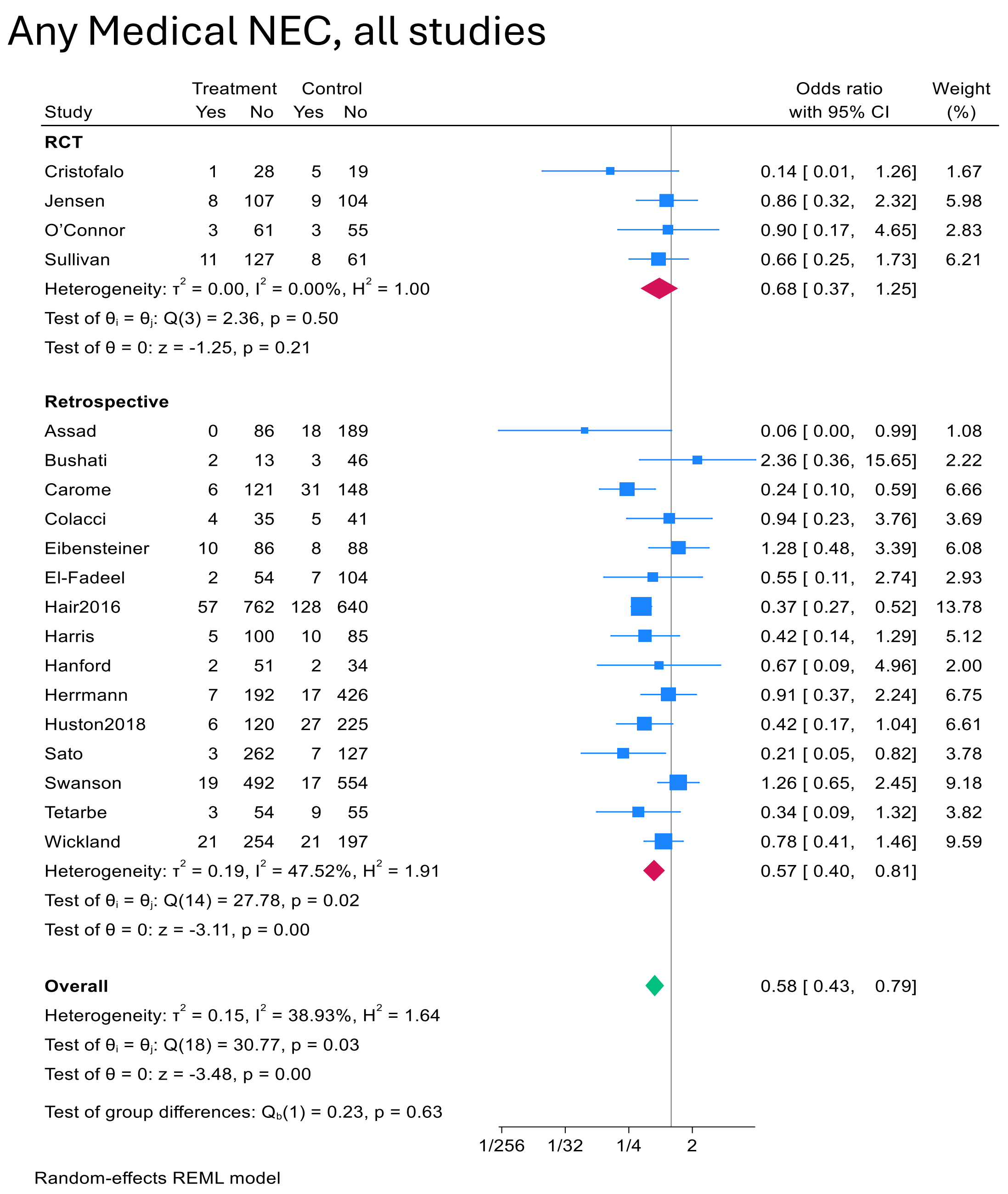

Figure S2. Associations of an EHMD vs. cow milk-containing diet with medical NEC among low birthweight infants (N = 6,708). Forest plots were generated based on random-effects meta-analysis using the Sidik-Jonkman method for between-study variance estimation—CI, confidence interval; CMD+F, cow milk containing diet which may include infant formula; EHMD, exclusive human milk diet; NEC, necrotizing enterocolitis
