# Supplementary material for: Human Milk Fortification and Necrotizing Enterocolitis in Very Low Birthweight Infants: State of Evidence and Systematic Review with Meta-Analysis
Source: Nutrients. 2025 Oct 28;17(21):3384. doi: 10.3390/nu17213384 (PMC12609769; doi:10.3390/nu17213384)
Supplement: Supplementary file 1 [file nutrients-17-03384-s001.zip › nutrients-3950813-supplementary/Table S1 SearchStrategy.pdf]

Table S1. Search Strategy for PubMed, Web of Science, and Scopus

Database	Search terms
**PubMed**	1. "Milk*"[Mesh] OR "Milk, Human"[Mesh] OR "Infant Food"[Mesh] OR "Infant Formula"[Mesh] OR "Food, Fortified*"[Mesh] OR "human milk"[tiab] OR "breast milk"[tiab] OR breastmilk[tiab] OR "donor human milk"[tiab] OR "human milk fortifier"[tiab] OR "exclusive human milk*"[tiab] OR "enteral feeding"[tw]
	2. "Infant, Premature, Diseases"[Mesh] OR "Enterocolitis, Necrotizing"[Mesh] OR "necrotizing enterocolitis" [tw] OR "medical NEC" [tw] OR "surgical NEC" [tw] OR "feeding intolerance" [tw] OR "feeding interruption" [tw]
	3. "Humans"[Mesh] OR "Female"[Mesh] OR "Male"[Mesh] OR "Infant, Premature"[Mesh] OR "Infant, Extremely Low Birth Weight"[Mesh] OR "Infant, Very Low Birth Weight"[Mesh] OR "Infant, Newborn"[Mesh] OR "premature infant*"[tw] OR "preterm infant*"[tw] OR "low birthweight"[tw]
	#1 AND #3 AND #2
	5. "Retrospective Studies"[Mesh] OR "Epidemiologic Studies"[Mesh] OR "Clinical Trial" [Publication Type] OR "Randomized Controlled Trial" [Publication Type] OR "Published Erratum" [Publication Type] OR "retrospective stud*"[tiab] OR "controlled trial"[tiab] OR "randomized clinical trial"[tiab] OR "observational study"[tiab] OR "multi-center retrospective cohort study"[tiab]
	**#1 AND #3 AND #2 AND #5**
**Web of Science**	1. TS=("Milk" OR "Infant Food" OR "Infant Formula" OR "human milk" OR "breast milk" OR (mothers NEAR/3 milk) OR "breast feeding" OR "donor human milk" OR "donor breast milk" OR "human milk fortifier" OR "exclusive human milk" OR "enteral feeding")
	2. TS=("necrotizing enterocolitis" OR "medical NEC" OR "surgical NEC" OR "feeding intolerance" OR "feeding interruption")
	3. TS=(Humans OR Female OR Male OR "Extremely Low Birth Weight" OR "infant, extremely low birthweight" OR "Very Low Birth Weight" OR "infant, very low birthweight" OR "Newborn Infant" OR "premature infant" OR "preterm infant" OR "low birthweight")
	**#1 AND #2 AND #3**
**Scopus**	(TITLE-ABS-KEY(Milk OR "Milk, Human" OR "Infant Food" OR "Infant Formula" OR "Food, Fortified" OR "human milk" OR "breast milk" OR breastmilk OR "donor human milk" OR "human milk fortifier" OR "exclusive human milk" OR "enteral feeding"))
	(TITLE-ABS-KEY("Infant, Premature, Diseases" OR "Enterocolitis, Necrotizing" OR "necrotizing enterocolitis" OR "medical NEC" OR "surgical NEC" OR "feeding intolerance" OR "feeding interruption" OR death OR survival))
	(TITLE-ABS-KEY(Humans OR Female OR Male OR "Infant, Premature" OR "Infant, Extremely Low Birth Weight" OR "Infant, Very Low Birth Weight" OR "Infant, Newborn" OR "premature infant" OR "preterm infant" OR "low birthweight"))
	(TITLE-ABS-KEY("Retrospective Studies" OR "Epidemiologic Studies" OR "Clinical Trial" OR "Randomized Controlled Trial" OR "Published Erratum" OR "retrospective study" OR "controlled trial" OR "randomized clinical trial" OR "observational study" OR "multi-center retrospective cohort study"))
	**#1 AND #2 AND #3 AND #4**
