# Supplementary material for: Human Milk Fortification and Necrotizing Enterocolitis in Very Low Birthweight Infants: State of Evidence and Systematic Review with Meta-Analysis
Source: Nutrients. 2025 Oct 28;17(21):3384. doi: 10.3390/nu17213384 (PMC12609769; doi:10.3390/nu17213384)
Supplement: Supplementary file 1 [file nutrients-17-03384-s001.zip › nutrients-3950813-supplementary/Table S2.pdf]

Table S2. Excluded studies (n=82)

References	Reason for Exclusion
1-45	Wrong intervention (n=45)
46-63	Wrong study design (n=18)
64-72	Wrong comparator (n=9)
73-76	Wrong outcomes (n=4)
77	Not in English (n=1)
78	Wrong publication type (n=1)
79-82	Data available in another included publication (n=4)

References

1. Zachariassen G, Fenger-Gron J. Preterm dietary study: meal frequency, regurgitation and the surprisingly high use of laxatives among formula-fed infants following discharge. *Acta Paediatr.* 2014;103(3):e116-122.
2. Adhisivam B, Kohat D, Tanigasalam V, Bhat V, Plakkal N, Palanivel C. Does fortification of pasteurized donor human milk increase the incidence of necrotizing enterocolitis among preterm neonates? A randomized controlled trial. *J Matern Fetal Neonatal Med.* 2019;32(19):3232-3237.
3. Alyahya W, Simpson J, Garcia AL, Mactier H, Young D, Edwards CA. Association between Early Feeding Patterns and Neonatal Outcomes in Very Preterm Infants: A Retrospective Cohort Study. *Neonatology.* 2023;120(1):71-80.
4. Barr PA, Mally PV, Caprio MC. Standardized Nutrition Protocol for Very Low-Birth-Weight Infants Resulted in Less Use of Parenteral Nutrition and Associated Complications, Better Growth, and Lower Rates of Necrotizing Enterocolitis. *JPEN J Parenter Enteral Nutr.* 2019;43(4):540-549.
5. Abramson J, Szatkowski L, Bains M, et al. Effects of implementation of a care bundle on rates of necrotising enterocolitis and own mother's milk feeding in the East Midlands: protocol for a mixed methods impact and process evaluation study. *BMJ Open.* 2024;14(5):e078633.
6. Belling-Dierks F, Glaser K, Wirbelauer J, Rücker V, Frieauff E. Does rapid enteral feeding increase intestinal morbidity in very low birth weight infants? A retrospective analysis. *J Matern Fetal Neonatal Med.* 2017;30(22):2690-2696.
7. Berkhout DJC, Klaassen P, Niemarkt HJ, et al. Risk Factors for Necrotizing Enterocolitis: A Prospective Multicenter Case-Control Study. *Neonatology.* 2018;114(3):277-284.
8. Bharadwaj N, Panigrahy N, Bagga N, Chirla DK. Human Milk-Derived Fortifier as Rescue Therapy in Very Preterm Infants Intolerant to Cow's Milk-Derived Fortifier. *Indian J Pediatr.* 2022;89(11):1131-1133.
9. Butler TJ, Szekely LJ, Grow JL. A standardized nutrition approach for very low birth weight neonates improves outcomes, reduces cost and is not associated with

increased rates of necrotizing enterocolitis, sepsis or mortality. *J Perinatol*. 2013;33(11):851-857.
10. Cakmak Celik F, Aygun C, Cetinoglu E. Does early enteral feeding of very low birth weight infants increase the risk of necrotizing enterocolitis? *Eur J Clin Nutr*. 2009;63(4):580-584.
11. Cañizo Vázquez D, Salas García S, Izquierdo Renau M, Iglesias-Platas I. Availability of Donor Milk for Very Preterm Infants Decreased the Risk of Necrotizing Enterocolitis without Adversely Impacting Growth or Rates of Breastfeeding. *Nutrients*. 2019;11(8).
12. Chinnappan A, Sharma A, Agarwal R, Thukral A, Deorari A, Sankar MJ. Fortification of Breast Milk With Preterm Formula Powder vs Human Milk Fortifier in Preterm Neonates: A Randomized Noninferiority Trial. *JAMA Pediatr*. 2021;175(8):790-796.
13. Christensen RD, Lambert DK, Gordon PV, Baer VL, Gerday E, Henry E. Neonates presenting with bloody stools and eosinophilia can progress to two different types of necrotizing enterocolitis. *J Perinatol*. 2012;32(11):874-879.
14. Colaizy TT, Poindexter BB, McDonald SA, et al. Neurodevelopmental Outcomes of Extremely Preterm Infants Fed Donor Milk or Preterm Infant Formula: A Randomized Clinical Trial. *Jama*. 2024;331(7):582-591.
15. Corpeleijn WE, de Waard M, Christmann V, et al. Effect of Donor Milk on Severe Infections and Mortality in Very Low-Birth-Weight Infants: The Early Nutrition Study Randomized Clinical Trial. *JAMA Pediatr*. 2016;170(7):654-661.
16. Corpeleijn WE, Kouwenhoven SM, Paap MC, et al. Intake of own mother's milk during the first days of life is associated with decreased morbidity and mortality in very low birth weight infants during the first 60 days of life. *Neonatology*. 2012;102(4):276-281.
17. Cortez J, Makker K, Kraemer DF, Neu J, Sharma R, Hudak ML. Maternal milk feedings reduce sepsis, necrotizing enterocolitis and improve outcomes of premature infants. *J Perinatol*. 2018;38(1):71-74.
18. D'Costa R, Fucile S PhD OT, Dickson BR, Gallipoli A, Dow Ke Md F. Benefits of a Standardized Enteral Feeding Protocol on the Nutrition and Health Outcomes of Very Low Birth Weight Preterm Infants. *Can J Diet Pract Res*. 2022;83(3):147-150.
19. Dritsakou K, Liosis G, Valsami G, Polychronopoulos E, Skouroliakou M. Improved outcomes of feeding low birth weight infants with predominantly raw human milk versus donor banked milk and formula. *J Matern Fetal Neonatal Med*. 2016;29(7):1131-1138.
20. Fortmann I, Marißen J, Siller B, et al. Lactobacillus Acidophilus/Bifidobacterium Infantis Probiotics Are Beneficial to Extremely Low Gestational Age Infants Fed Human Milk. *Nutrients*. 2020;12(3).
21. Gates A, Thompson AB, Marin T, Waller JL, Patel J, Stansfield BK. Novel multinutrient human milk-based human milk fortifier promotes growth and tolerance in premature infants. *JPEN J Parenter Enteral Nutr*. 2022;46(4):817-827.
22. Gephart SM, Quinn MC. Relationship of Necrotizing Enterocolitis Rates to Adoption of Prevention Practices in US Neonatal Intensive Care Units. *Adv Neonatal Care*. 2019;19(4):321-332.

23. Gregory KE. Clinical predictors of necrotizing enterocolitis in premature infants. *Nurs Res*. 2008;57(4):260-270.
24. Hällström M, Koivisto AM, Janas M, Tammela O. Frequency of and risk factors for necrotizing enterocolitis in infants born before 33 weeks of gestation. *Acta Paediatr*. 2003;92(1):111-113.
25. Hammond PJ, Flett ME, De La Hunt M. Fulminant necrotising enterocolitis immediately following change to low birth weight formula feeds. *Eur J Pediatr Surg*. 2008;18(3):185-187.
26. Hanson C, Sundermeier J, Dugick L, Lyden E, Anderson-Berry AL. Implementation, process, and outcomes of nutrition best practices for infants <1500 g. *Nutr Clin Pract*. 2011;26(5):614-624.
27. Hemmati F, Ghassemzadeh M. The Effect of Oral Protein Supplementation on the Growth of Very Low Birth Weight Preterm Infants Admitted to the Neonatal Intensive Care Unit: A Randomized Clinical Trial. *J Mother Child*. 2023;27(1):21-29.
28. Hogewind-Schoonenboom JE, Rövekamp-Abels LWW, de Wijs-Meijler DPM, et al. The Effect of Maternal Milk on Tolerance and Growth in Premature Infants: A Hypothesis-generating Study. *J Pediatr Gastroenterol Nutr*. 2017;64(6):971-974.
29. Kanmaz HG, Mutlu B, Canpolat FE, et al. Human milk fortification with differing amounts of fortifier and its association with growth and metabolic responses in preterm infants. *J Hum Lact*. 2013;29(3):400-405.
30. Klingenberg C, Muraas FK, Isaksen CE, Nilsen T, Torgersen M, Melum-Hansen C. Growth and neurodevelopment in very preterm infants receiving a high enteral volume-feeding regimen - a population-based cohort study. *J Matern Fetal Neonatal Med*. 2019;32(10):1664-1672.
31. Kombo L, Smith J, Van Wyk L. Somatic Growth of Enteral-Only Fed Extremely Low Birth Weight Infants in a Resource-Restricted Setting. *J Trop Pediatr*. 2021;67(1).
32. Le VT, Klebanoff MA, Talavera MM, Slaughter JL. Transient effects of transfusion and feeding advances (volumetric and caloric) on necrotizing enterocolitis development: A case-crossover study. *PLoS One*. 2017;12(6):e0179724.
33. Ma XW, Fan WQ. Earlier Nutrient Fortification of Breastmilk Fed LBW Infants Improves Jaundice Related Outcomes. *Nutrients*. 2020;12(7).
34. Masoli D, Mena P, Dominguez A, et al. Growth of Very Low Birth Weight Infants Who Received a Liquid Human Milk Fortifier: A Randomized, Controlled Multicenter Trial. *J Pediatr Gastroenterol Nutr*. 2022;74(3):424-430.
35. Moya F, Sisk PM, Walsh KR, Berseth CL. A new liquid human milk fortifier and linear growth in preterm infants. *Pediatrics*. 2012;130(4):e928-935.
36. Murthy S, Parker PR, Gross SJ. Low rate of necrotizing enterocolitis in extremely low birth weight infants using a hospital-based preterm milk bank. *J Perinatol*. 2019;39(1):108-114.
37. Parm Ü, Metsvaht T, Ilmoja ML, Lutsar I. Gut colonization by aerobic microorganisms is associated with route and type of nutrition in premature neonates. *Nutr Res*. 2015;35(6):496-503.

38. Patel S, Chaudhari M, Kadam S, Rao S, Patole S. Standardized feeding and probiotic supplementation for reducing necrotizing enterocolitis in preterm infants in a resource limited set up. *Eur J Clin Nutr.* 2018;72(2):281-287.
39. Peng W, Han J, Li S, et al. The Association of Human Milk Feeding With Short-Term Health Outcomes Among Chinese Very/Extremely Low Birth Weight Infants. *J Hum Lact.* 2022;38(4):670-677.
40. Reali A, Greco F, Marongiu G, et al. Individualized fortification of breast milk in 41 Extremely Low Birth Weight (ELBW) preterm infants. *Clin Chim Acta.* 2015;451(Pt A):107-110.
41. Rochow N, Fusch G, Ali A, et al. Individualized target fortification of breast milk with protein, carbohydrates, and fat for preterm infants: A double-blind randomized controlled trial. *Clin Nutr.* 2021;40(1):54-63.
42. Sisk PM, Lambeth TM, Rojas MA, et al. Necrotizing Enterocolitis and Growth in Preterm Infants Fed Predominantly Maternal Milk, Pasteurized Donor Milk, or Preterm Formula: A Retrospective Study. *Am J Perinatol.* 2017;34(7):676-683.
43. Stefanescu BM, Gillam-Krakauer M, Stefanescu AR, Markham M, Kosinski JL. Very low birth weight infant care: adherence to a new nutrition protocol improves growth outcomes and reduces infectious risk. *Early Hum Dev.* 2016;94:25-30.
44. Trang S, Zupancic JAF, Unger S, et al. Cost-Effectiveness of Supplemental Donor Milk Versus Formula for Very Low Birth Weight Infants. *Pediatrics.* 2018;141(3).
45. Yigit S, Akgoz A, Memisoglu A, Akata D, Ziegler EE. Breast milk fortification: effect on gastric emptying. *J Matern Fetal Neonatal Med.* 2008;21(11):843-846.
46. Fleig L, Hagan J, Lee ML, Abrams SA, Hawthorne KM, Hair AB. Growth outcomes of small for gestational age preterm infants before and after implementation of an exclusive human milk-based diet. *J Perinatol.* 2021;41(8):1859-1864.
47. Ganapathy V, Hay JW, Kim JH. Costs of necrotizing enterocolitis and cost-effectiveness of exclusively human milk-based products in feeding extremely premature infants. *Breastfeed Med.* 2012;7(1):29-37.
48. Hair AB, Hawthorne KM, Chetta KE, Abrams SA. Human milk feeding supports adequate growth in infants ≤ 1250 grams birth weight. *BMC Res Notes.* 2013;6.
49. Hair AB, Rechtman DJ, Lee ML, Niklas V. Beyond Necrotizing Enterocolitis: Other Clinical Advantages of an Exclusive Human Milk Diet. *Breastfeed Med.* 2018;13(6):408-411.
50. Underwood MA, Kalanetra KM, Bokulich NA, et al. Prebiotic oligosaccharides in premature infants. *J Pediatr Gastroenterol Nutr.* 2014;58(3):352-360.
51. Vardar G, Ozdil M, Tufekci S. Awareness or neglecting the diagnosis of cow milk protein allergy in the neonatal period. *Asia Pac J Clin Nutr.* 2023;32(2):257-264.
52. Osmanova M, Müller MJ, Habisch B, Hippe A, Seeliger S. Nutrition of Infants with Very Low Birth Weight using Human and Bovine Based Milk Fortifier: Benefits and Costs. *Neonatal and Pediatric Medicine.* 2021;7(S10).
53. Grace E, Hilditch C, Gomersall J, Collins CT, Rumbold A, Keir AK. Safety and efficacy of human milk-based fortifier in enterally fed preterm and/or low birthweight infants: a systematic review and meta-analysis. *Arch Dis Child Fetal Neonatal Ed.* 2021;106(2):137-142.

54. van Katwyk S, Ferretti E, Kumar S, et al. Economic Analysis of Exclusive Human Milk Diets for High-Risk Neonates, a Canadian Hospital Perspective. *Breastfeed Med*. 2020;15(6):377-386.
55. Hampson G, Roberts SLE, Lucas A, Parkin D. An economic analysis of human milk supplementation for very low birth weight babies in the USA. *BMC Pediatr*. 2019;19(1):337.
56. Aoki M, Motoichirou S, Mizuno K, Nakano Y, Asai H, Hawthorne KM. First Use in Japan of Exclusive Human Milk Diet: Case Report Series. . *Ann Clin Case Rep*. 2020;5:1877.
57. Lofiego P, Samedi V, Rai SE. Severe feeding intolerance in extremely preterm neonates successfully treated with human milk derived human milk fortifier: A case series. *Journal of Clinical Neonatology.* 2021;10(4).
58. Reyes SM, Moore JB, Lee ML, Ferry J, Elliott MJ. Associations of an Exclusive Human Milk Diet with Morbidity and Mortality in ELBW Infants Born Weighing <750 Grams: An Individual Participant Data Meta-analysis. *Neonatology Today* 2023;18(4):3-23.
59. Scholz SM, Greiner W. An exclusive human milk diet for very low birth weight newborns-A cost-effectiveness and EVPI study for Germany. *PLoS One*. 2019;14(12):e0226496.
60. Lucas A, Assad M, Sherman J, Boscardin J, Abrams S. Safety of Cow's Milk-Derived Fortifiers Used with an All-Human Milk Base Diet in Very Low Birthweight Preterm Infants. *Neonatology Today.* 2020;15(7):3-16.
61. Vasu V, Mulla S, Pandya A, Card D, Shearer MJ, Clarke P. Late-onset vitamin K deficiency bleeding in an extremely preterm infant fed an exclusively human milk-based diet. *J Thromb Haemost.* 2023.
62. Philip RK, Romeih E, Bailie E, et al. Exclusive Human Milk Diet for Extremely Premature Infants: A Novel Fortification Strategy That Enhances the Bioactive Properties of Fresh, Frozen, and Pasteurized Milk Specimens. *Breastfeed Med*. 2023;18(4):279-290.
63. Abrams SA, Schanler RJ, Lee ML, Rechtman DJ. Greater mortality and morbidity in extremely preterm infants fed a diet containing cow milk protein products. *Breastfeed Med*. 2014;9(6):281-285.
64. Ford SL, Lohmann P, Preidis GA, et al. Improved feeding tolerance and growth are linked to increased gut microbial community diversity in very-low-birth-weight infants fed mother's own milk compared with donor breast milk. *Am J Clin Nutr*. 2019;109(4):1088-1097.
65. Huston R, Lee M, Rider E, et al. Early fortification of enteral feedings for infants <1250 grams birth weight receiving a human milk diet including human milk based fortifier. *J Neonatal Perinatal Med.* 2020;13(2):215-221.
66. Hair AB, Blanco CL, Moreira AG, et al. Randomized trial of human milk cream as a supplement to standard fortification of an exclusive human milk-based diet in infants 750-1250 g birth weight. *J Pediatr.* 2014;165(5):915-920.
67. Visuthranukul C, Abrams SA, Hawthorne KM, Hagan JL, Hair AB. Premature small for gestational age infants fed an exclusive human milk-based diet achieve catch-up

growth without metabolic consequences at 2 years of age. . *Arch Dis Child Fetal Neonatal Ed.* 2019;104(3):F242-F247.
68. Bergner EM, Shypailo R, Visuthranukul C, et al. Growth, Body Composition, and Neurodevelopmental Outcomes at 2 Years Among Preterm Infants Fed an Exclusive Human Milk Diet in the Neonatal Intensive Care Unit: A Pilot Study. *Breastfeed Med.* 2020;15(5):304-311.
69. Rahman A, Kase JS, Murray YL, Parvez B. Neurodevelopmental Outcome of Extremely Low Birth Weight Infants Fed an Exclusive Human Milk Diet Is Not Affected by Growth Velocity. *Breastfeed Med.* 2020;15(6):362-369.
70. Chetta KE, Hair AB, Hawthorne KM, Abrams SA. Serum phosphorus levels in premature infants receiving a donor human milk derived fortifier. *Nutrients.* 2015;7(4):2562-2573.
71. Salas AA, Gunawan E, Nguyen K, et al. Early Human Milk Fortification in Infants Born Extremely Preterm: A Randomized Trial. *Pediatrics.* 2023;152(3).
72. Lambert DK, Christensen RD, Baer VL, et al. Fulminant necrotizing enterocolitis in a multihospital healthcare system. *J Perinatol.* 2012;32(3):194-198.
73. Hair AB, Patel AL, Kiechl-Kohlendorfer U, et al. Neurodevelopmental outcomes of extremely preterm infants fed an exclusive human milk-based diet versus a mixed human milk + bovine milk-based diet: a multi-center study. *J Perinatol.* 2022;42(11):1485-1488.
74. Hopperton KE, O'Connor DL, Bando N, et al. Nutrient Enrichment of Human Milk with Human and Bovine Milk-Based Fortifiers for Infants Born <1250 g: 18-Month Neurodevelopment Follow-Up of a Randomized Clinical Trial. *Curr Dev Nutr.* 2019;3(12).
75. Ackley D, Wang H, D'Angio CT, Meyers J, Young BE. Human milk-derived fortifiers are linked with feed extension due to Hypoglycemia in infants <1250 g or <30 weeks: a matched retrospective chart review. *J Perinatol.* 2023;43(5):624-628.
76. Holzapfel LF, Hair AB, Preidis GA, et al. Fecal Elastase in Preterm Infants to Predict Growth Outcomes. *J Pediatr Gastroenterol Nutr.* 2023;76(2):206-212.
77. Wang L, Zhao XP, Liu HJ, et al. [Evidence-based standardized nutrition protocol can shorten the time to full enteral feeding in very preterm/very low birth weight infants]. *Zhongguo Dang Dai Er Ke Za Zhi.* 2022;24(6):648-653.
78. Jensen GB, Ahlsson F, Domellöf M, Elfvin A, Naver L, Abrahamsson T. Nordic study on human milk fortification in extremely preterm infants: a randomised controlled trial-the N-forte trial. *BMJ Open.* 2021;11(11):e053400.
79. Delaney Manthe E, Perks PH, Swanson JR. Team-Based Implementation of an Exclusive Human Milk Diet. *Adv Neonatal Care.* 2019;19(6):460-467.
80. Chang MR, Tetarbe M, Barton L, Ramanathan R, Cayabyab R. Transient Hypoglycemia and Biochemical Differences in Infants Less Than 1,250 G at Birth Fed Human Milk with Human Milk-Derived Fortifier versus Cow Milk-Derived Fortifier. *Am J Perinatol.* 2023.
81. Uthaya S, Jeffries S, Andrzejewska I, Vasu V, Embleton ND, Modi N. Randomised controlled trial of human derived breast milk fortifier versus bovine milk fortifier on body composition in very preterm babies. *Early Hum Dev.* 2022;171:105619.

82. Huston RK, Markell AM, McCulley EA, et al. Decreasing Necrotizing Enterocolitis and Gastrointestinal Bleeding in the Neonatal Intensive Care Unit. *ICAN: Infant, Child, & Adolescent Nutrition*. 2014;6(2):86-93.
