# Supplementary material for: Human Milk Fortification and Necrotizing Enterocolitis in Very Low Birthweight Infants: State of Evidence and Systematic Review with Meta-Analysis
Source: Nutrients. 2025 Oct 28;17(21):3384. doi: 10.3390/nu17213384 (PMC12609769; doi:10.3390/nu17213384)
Supplement: Supplementary file 1 [file nutrients-17-03384-s001.zip › nutrients-3950813-supplementary/Tables S3.pdf]

Table S2. Risk of bias assessment for RCTs

Citation	**Overall**	Randomization	Deviation from intended intervention	Missing outcome data	Measurement of the outcome	Selection of the reported result
Cristofalo 2013	**LR**	LR	LR	LR	LR	LR
Embleton 2023	**SC**	SC	LR	LR	LR	LR
Jensen 2023	**LR**	LR	LR	LR	LR	LR
O'Connor 2018	**LR**	LR	LR	LR	LR	LR
Sullivan 2010	**LR**	LR	LR	LR	LR	LR

LR low risk ; SC some concerns ; HR high risk
