# Supplementary material for: Human Milk Fortification and Necrotizing Enterocolitis in Very Low Birthweight Infants: State of Evidence and Systematic Review with Meta-Analysis
Source: Nutrients. 2025 Oct 28;17(21):3384. doi: 10.3390/nu17213384 (PMC12609769; doi:10.3390/nu17213384)
Supplement: Supplementary file 1 [file nutrients-17-03384-s001.zip › nutrients-3950813-supplementary/Tables S4.pdf]

Table S3. Risk of bias assessment for observational studies

**Citation**	**Overall**	Confounding	Selection of participants	Classification of exposures	Departures from intended exposures	Missing data	Measurement of outcomes	Selection of reported results
Assad 2013	**MR**	**MR**	LR	MR	LR	LR	LR	MR
Bushati 2021	**SR**	SR	LR	LR	MR	LR	LR	MR
Carome 2020	**SR**	MR	LR	LR	SR	LR	LR	MR
Colacci 2017	**SR**	MR	LR	MR	LR	LR	MR	MR
Eibensteiner 2019	**MR**	MR	LR	LR	LR	LR	MR	LR
El-Fadeel 2022	**SR**	MR	LR	MR	LR	LR	MR	MR
Hair 2016	**SR**	MR	MR	LR	MR	LR	MR	MR
Hanford 2021	**MR**	MR	LR	LR	LR	LR	LR	MR
Harris 2024	**SR**	SR	LR	MR	MR	LR	MR	NI
Herrmann 2014	**CR**	SR	MR	MR	LR	MR	MR	LR
Huston 2018	**CR**	MR	SR	SR	MR	LR	MR	MR
Sato 2020	**CR**	SR	MR	LR	SR	SR	MR	MR
Swanson 2023	**SR**	SR	LR	MR	MR	LR	MR	NI
Tetarbe 2024	**SR**	MR	MR	LR	MR	MR	MR	MR
Wickland 2022	**MR**	MR	LR	LR	LR	LR	MR	MR

LR low risk ; MR moderate risk ; SR serious risk ; CR critical risk ; NI no information
